# Plant Compounds Inhibit the Growth of W12 Cervical Precancer Cells Containing Episomal or Integrant HPV DNA; Tanshinone IIA Synergizes with Curcumin in Cervical Cancer Cells

**DOI:** 10.3390/v17010055

**Published:** 2024-12-31

**Authors:** Linda Saxe Einbond, Jing Zhou, Kunhui Huang, Mario R. Castellanos, Emeka Mbazor, Michael Balick, Hongbao Ma, James A. DeVoti, Stephen Redenti, Hsan-au Wu

**Affiliations:** 1 Center for Plants, People and Culture, The New York Botanical Garden, New York, NY 10458, USA; mbalick@nybg.org; 2 Lehman College and the Graduate Center, City University of New York, New York, NY 10468, USA; zhouzhou0923@hotmail.com (J.Z.); kunhui.huang@lehman.cuny.edu (K.H.); embazor@gmail.com (E.M.); stephen.redenti@lehman.cuny.edu (S.R.); 3 Department of Rehabilitation and Regenerative Medicine, College of Physicians and Surgeons, Columbia University, HHSC-1518, 701 W. 168th Street, New York, NY 10032, USA; mahongbao2022@gmail.com (H.M.); hsanau.wu@gmail.com (H.-a.W.); 4 Staten Island University Hospital, Northwell Health, New York, NY 10305, USA; mario_md@yahoo.com; 5 Innovene Therapeutics, Jersey City, NJ 07302, USA; 6 The Feinstein Institutes for Medical Research, Northwell Health, Manhasset, NY 11030, USA; jdevoti@northwell.edu

**Keywords:** bioelectricity, curcumin, episomal, precancer, tanshinone IIA, molecular docking

## Abstract

This study explores the effects of plant compounds on human papillomavirus (HPV)-induced W12 cervical precancer cells and bioelectric signaling. The aim is to identify effective phytochemicals, both individually and in combination, that can prevent and treat HPV infection and HPV associated cervical cancer. Phytochemicals were tested using growth inhibition, combination, gene expression, RT PCR, and molecular docking assays. W12 cells, derived from a cervical precancerous lesion, contain either episomal or integrated HPV16 DNA. Several compounds, including digoxin, tanshinone IIA, dihydromethysticin and carrageenan, as well as fractions of turmeric, ginger and pomegranate inhibited the growth of W12 precancer and cervical cancer cells. Curcumin and tanshinone IIA were the most active and relatively nontoxic compounds. RT-PCR analysis showed that tanshinone IIA activated the expression of p53, while repressing the expression of HPV16 E1, E2, E4, E6, and E7 viral transcripts in W12 (type 1 and 2) integrant cells. In addition, curcumin synergized with tanshinone IIA in HeLa cells. Molecular docking studies suggested tanshinone IIA and curcumin bind to the Na^+^/K^+^-ATPase ion channel, with curcumin binding with higher affinity. Our findings highlight the potential of these multifaceted phytochemicals to prevent and treat HPV-induced cervical cancer, offering a promising approach for combinatorial therapeutic intervention.

## 1. Introduction

To explore potential treatments for cervical cancer prevention and therapy, we investigated the efficacy of phytochemicals, alone and in combination, in use in traditional medicine (TCM and Ayurvedic) [1].

Cervical cancer is the fourth most common female cancer worldwide (2020) [2], and is caused by persistent human papillomavirus (HPV) infection. Prophylactic vaccines can prevent common high-risk HPV genotypes that induce cervical cancer. However, these vaccines are not always available in developing countries, and do not always protect against every strain of oncogenic HPV. Moreover, women who are already infected do not benefit from these vaccines. Currently, there is no approved medication to treat cervical precancer or HPV infection. Natural products are attractive since they act on multiple targets, are often readily available, and have a history of use in patients.

After HPV infection, the virus produces several viral proteins, E1, E2, E4, E5, E6, E7, L1 and L2. E1, E2, E4 and E5 function in replication [3], while E6 and E7 function in cell transformation [4]. The expression of HPV16 oncoproteins E6 and E7 is essential to develop and maintain the malignant state [4].

Studies have shown that curcumin [5], tanshinone IIA [6,7,8], ellagic acid [9], epigallocatechin gallate (EGCG) [10], and the botanical mixture TriCurin (curcumin:EGCG:resveratrol) [11] inhibit the growth of HPV(+) cervical cancer cells.

This study builds on our previous research with TriCurin, which highlights the synergistic effects of plant polyphenols [12]; our goal is to identify additional promising phytochemicals and their combinations for potential use against HPV. Plants have developed unique phytochemicals as defense mechanisms against various pathogens, including viruses. This research aims to leverage these to develop new, relatively non-toxic, treatments for HPV-induced diseases.

The herbs and compounds were selected for their antiviral, anti-inflammatory and antioxidant properties, as well as their diverse mechanisms of action. Selections were made from traditional medicine systems (TCM, Ayurvedic, Pacific Island remedies, and Western herbalism) and modern phytochemical research.

Among the phytochemicals tested were tanshinone IIA from Danshen, derived from *Salvia* *miltiorrhiza* var. *miltiorrhiza*, dihydromethysticin from kava, actein from black cohosh, carrageenan from red seaweed, carnosic acid from rosemary, digoxin from foxglove, and enriched extracts or fractions of turmeric, pomegranate, ginger, kava, milk thistle, and an extract of blueberry. This selection aims to target HPV through multiple pathways.

To gain insight into the mode of action of the phytochemicals, we explored the growth inhibitory effects of the most active phytochemicals on cervical keratinocytes derived from a precancerous lesion (W12; CIN grade 1). W12E cells contain full-length HPV16 episomes and W12I contain integrated HPV16 DNA. W12I clones can further be categorized into type 1 integration (3–5 genomes) and type 2 integration (30–60 genomes) [13]. In this study, we examined the effect of tanshinone IIA on the expression of p53 and HPV16 mRNAs, in W12 cells, using RT-PCR.

Since cancer affects multiple genes, the optimal treatment may require mixtures. To identify effective treatments, we tested the growth inhibitory activity of combinations of the most potent phytochemicals. Our study indicates tanshinone IIA synergizes with curcumin. Recent advances in bioelectric signaling underscore the ability of ion channel molecules, such as the Na^+^/K^+^-ATPase, to modulate the development and progression of cancer. To better understand the mode of action of curcumin and tanshinone IIA, alone and in combination, we assessed their binding to the Na^+^/K^+^-ATPase, using molecular docking. Both compounds bind; curcumin binds with high affinity, comparable to that of digitoxin; while tanshinone IIA exhibits weaker binding. Our findings suggest the potential of these phytochemicals to prevent and treat cervical cancer.

## 2. Materials and Methods

### 2.1. Materials

The following herbal enriched extracts or fractions that we tested were kindly provided by Naturex (Avignon, France): turmeric (*Curcuma longa* L.) root (95% curcuminoids, Item #: 140500; the extract contained approximately 74% curcumin (diferuloylmethane): typical range: curcumin (72–75%); bisdemethoxycurcumin (3–4%); demethoxycurcumin (16–17%) (HPLC: Figure S1D); pomegranate (*Punica granatum* L.) fruit (44% ellagic acid, Item #: 140904); milk thistle (*Silybum marianum* (L.) Gaertn.) seed (80.4% silymarin, Item #: 140200); ginger (*Zingiber officinale* Roscoe) root soft extract (20.5% total pungent compounds calculated as gingerols and shogaols, Item #: 143010); licorice (*Glycyrrhiza glabra*) root (16.99% glycyrrhizin; Item #: 161596); Boswellia (*Boswellia serrata*; 68.1% boswellic acids, Item #: 161136); blueberry (*Vaccinium* species) fruit (Strength: 5:1, Item #: 161118); kava (*Piper methysticum* G. Forst; 31.2% kavalactones, Item #: 332679); (Technical data sheets and Certificates of analysis: most important extracts; Supplementary Materials: Tables S6–S10).

Curcumin powder (95%; 71.86% curcumin; 89.88% total curcuminoids; 96.88% total curcuminoid complex consisting of curcumin, desmethoxycurcumin, bis-demethoxycurcumin and volatile oils of turmeric rhizome (Dolcas Biotech LLC., Landing, NJ, USA) was purchased from PCCA (Houston, TX, USA)).

The following compounds were purchased from Sigma (St. Louis, MO, USA): tanshinone IIA (≥97%); stilbene (≥96%,); carnosic acid from *Salvia rosmarinus* Spenn (≥91%;); lambda carrageenan (red seaweed: *Chondrus crispus* Stackh) plant mucopolysaccharide; alpha–kavain (≥95%); and digoxin (*Digitalis purpurea* L.; ≥95%). Dihydroxymethysticin (DHM) (≥85%) was purchased from Chromadex (Los Angeles, CA, USA); actein (>98%), from Planta Analytica (New Milford, CT, USA); and xanthochymol was the kind gift of Dr. E. Kennelly (Lehman College, New York, NY, USA). In the second set of W12 experiments, more dilute stock solutions were prepared. (Technical data sheets and Certificates of analysis: most important compounds; Supplementary Materials: Tables S11–S15).

The source of curcumin for the gene expression studies was as follows: curcumin (≥98% curcuminoid content) (CAS number 458-37-7) (Thermo Fisher Scientific, Waltham, MA, USA); prepared by Acros Organics (Geel, Belgium) for Thermo Fisher; stored at room temperature under nitrogen).

Figure S1A–D displays the structures of curcumin, tanshinone IIA and dihydromethysticin and an HPLC analysis of turmeric (95% curcuminoids).

### 2.2. Cell Culture: Cancer Cells

HeLa human cervical cancer cells were the kind gift of Dr. Bettie Steinberg (Northwell Health, New Hyde Park, NY, USA) and were obtained from ATCC. HT29 (p53 positive; similar to enterocytes from the small intestine) colon cancer cells were obtained from ATCC (Manassus, VA, USA). HeLa cells were grown in Dulbecco’s Modified Eagle’s medium (DMEM) (Gibco BRL Life Technologies, Inc., Rockville, MD, USA) containing 10% (*v*/*v*) fetal bovine serum (FBS) (Gibco BRL), plus Pen Strep (Gibco), while HT29 were maintained in McCoy’s media plus 10% FBS, plus Pen Strep (Gibco); 37 °C, 5% C02.

The online ATCC brochure “Maintaining High Standards in Cell Culture” describes the accessioning process, during which all ATCC cell lines undergo authentication tests [14].

### 2.3. Cell Culture: W12 Cells

W12 subclones W12E (episomal, 20850) and W12I (integrant: type 1: 20822, 201402; type 2: 20861, 20862) and J2 3T3 mouse embryo fibroblasts, were the kind gift of Dr. Paul Lambert (University of Wisconsin, Madison, WI, USA; 28 November 2016). The W12 cells are derived from a cervical intraepithelial lesion (CIN grade 1); the subclone 20850 contains approximately 5–100 extrachromosomal HPV16 genomes. After culture in vitro, the W12 cells generated the clones 20822 and 201402 containing 3 and 5 HPV16 genomes, respectively, that are recombined with the cellular DNA (type 1 integrated clones). In addition, the W12 cells generated clones 20861 and 20862, which contain many HPV16 genomes, 30 or 60 recombined viral genomes (viral genomes plus junction fragments), respectively, (type 2 integration) [13].

The W12 type 1 and 2 clones resemble the HPV DNA integration state of cancer derived cell lines, SiHa (1-2 HPV16 copies; CIN grade 2) and CaSki (500 HPV16 copies; CIN grade 3), respectively [13].

W12 clones were cultured to remain undifferentiated, as previously described [13]. Briefly, W12 cells were cultured on mitomycin C-treated J2 3T3 feeder cells in F medium (three parts F-12 medium); one part Dulbecco’s modified Eagle’s medium; 5% fetal bovine serum (FBS); insulin (5 μg/mL); cholera toxin (8.4 ng/mL); adenine (24 μg/mL), epidermal growth factor (10 ng/mL) and hydrocortisone (0.4 μg/mL). After 24 h, medium was changed to E medium (10 ng/mL epidermal growth factor in F medium). To passage, cells were rinsed using 0.02% EDTA solution and then treated with Trypsin/EDTA solution for 3 min. The ratio of W12 to feeder cells was estimated using microscopy analysis. The W12 cultures contained irradiated 3T3 (mitomycin C-treated J2 3T3 feeder) cells that are metabolically active, but not replicating. The ratio of feeder to W12 was approximately 1:20 to 1:100 (for 20861, 20862, 20822, 201402 cells) and about 1:10 to 1:50 for 20850 cells. The 201402 cells exhibited poor health and had a slow growth rate.

### 2.4. Proliferation Assay

Cells were seeded in 96 well plates at 1000 cells per well. After incubating for 24 h, phytochemicals were added. For the W12 assays, plant solutions were sonicated for 10 min each [15] to increase their solubility. This may degrade the structure of carrageenan [16]. For the combination experiments, plant solutions were sonicated for 10 min, two times, before addition to cell cultures [17]. The sensitivity of the various cell lines to herbal agents was assayed using the MTT assay [18] or the EZQUANT assay. Values were normalized to those of cells treated with vehicle controls.

For the EZQUANT assay, absorbance was read at 420 nM and corrected for the background values. IC_50_ values were estimated from the graphs, using a line for 50% cell proliferation. In the second and later sets of experiments on W12 cells, treatments were prepared from a lower concentration of stock solutions; the resulting IC_50_ values were lower.

### 2.5. Calculating the Combination Index

To assess possible synergistic effects, cells were treated with all combinations of 4 concentrations of each of the agents tested and a solvent control, as previously described [19].

The median effect principle was used to analyze the combination assays [19]. We used variable ratios of drugs and assumed mutually exclusive equations to determine the Combination Index (CI) [19]. IC_50_ values determined from the graphs were used to obtain combination index values; for agents 1 and 2: CI = [IC_50_ (agent 1 + agent 2)/(IC_50_ (agent 1 alone)] + [IC_50_ (agent 2 + agent 1)/IC_50_ (agent 2 alone)]. CI and its corresponding effects are as follows: >1.3, antagonism; 1.1–1.3, moderate antagonism; 0.9–1.1, additive effect; 0.8–0.9, slight synergism; 0.6–0.8, moderate synergism; <0.6, synergism.

### 2.6. RNA Extraction and Quantitative Real-Time PCR

Cells were seeded and cultured on 12 well plates at 2 × 10^5^ cells/well. Each drug concentration was performed with triplicates. At 0 h and 24 h, cells were collected and total RNA extraction was performed using TRIzol reagent (Thermo Fisher Scientific, Waltham, MA, USA). RNA concentration was measured using NanoDrop 2000. cDNA was synthesized using ProtoScript® First Strand cDNA Synthesis Kit (NEB Biolabs, Ipswich, MA, USA). GAPDH or b-actin was used as a housekeeping gene control for mRNA analysis. Quantitative real time PCR (qPCR) was performed using SYBR GreenER™ qPCR SuperMix (Life Technologies, Carlsbad, CA, USA) on a Bio-Rad system. Fold change 2^−∆∆C^ method was used to calculate mRNA abundance. Primer sequences used in qPCR are listed in Table S1. Primer efficiency was determined relative to that of the housekeeping genes (GAPDH or b-actin). Student’s *t* test was used to calculate the significance of the differences among the triplicate samples.

### 2.7. Gene Expression Analysis:

Gene expression analysis was performed, as previously described [12]. HeLa cells were treated with curcumin (32 µM) or control (DMSO) for 8 h. DMSO in all the results refers to the control condition, which has DMEM culture medium containing 0.28% of DMSO. The cells (1.5 × 10^5^ per well, in 24-well plates) were treated in triplicate in serum-free DMEM containing insulin-transferrin-sodium selenite (ITS). At the end of treatment, 200 µL of RLT buffer (Qiagen RNeasy Mini Kit, Qiagen Gmbh, Hilden, Germany) was added to each well and samples were frozen at −80 °C.

### 2.8. Docking Simulation:

The crystal structure of the Na^+^/K^+^-ATPase was obtained from the Protein Data Bank (PDB iD: 7DDI) (https://www.rcsb.org/structure/7DDI (accessed on 9 February 2024). The protein structure was prepared for docking and the binding was identified using the OEDocking (version 4.2.1) protein preparation tool SPRUCE (version 1.5.2) in the default parameters. The ligand structures were obtained from the PubChem (https://pubmed.ncbi.nlm.nih.gov/ (accessed on 9 February 2024)) database and prepared for the docking using OMEGA (version 4.1.2) of OpenEye toolkit.

Docking simulation: The docking of ligands to the Na^+^/K^+^-ATPase was performed using the FRED module from OEDOCKING (version 4.2.1) of OpenEye software (OpenEye Scientific Software, Inc., Santa Fe, NM, USA; https://www.eyesopen.com, release on 1 January 2023). The docking parameters and scoring function were default settings.

The docked poses were analyzed by the OEDocking in-house modules. The best docking pose for each ligand was chosen based on the ChemGauss4 scoring function, which is based on the analysis of interactions between protein and ligand, including the shape, hydrogen bonding and chelating interactions. The results of the docking and the potential hydrogen bonding were demonstrated by Chimera (Chimera 1.16, UCSF).

### 2.9. Statistical Analysis

Cell growth assays: Data are expressed as mean +/− standard deviation. The percentages of viable cells in control and treated samples were compared using the two-sample *t*-test (significance: *p* < 0.05).

Combination assays: A 2-tail Student’s *t*-test assuming two samples with equal variance, and a two-factor Analysis of Variance (ANOVA) based *t*-test for comparing 2 different means were used. Anova was utilized to estimate standard error (SE) of comparisons. In theory, the ANOVA based *t*-test is more accurate than the simple student’s *t*-test. Statisticsl significance was defined as *p* < 0.05 (Results: Section 3.3; Supplementary Materials: Tables S2–S5).

RT-PCR: Each primer pair was tested in triplicate.. Student’s *t*-test was used to identify significant differences in mRNA, Ct values; *p* values of less than 0.05 were considered statistically significant.

Gene expression analysis: p values were calculated using ANOVA KaleidaGraph, with post hoc tests performed using Tukey. Differentially expressed genes were selected from the KEGG pathway database (https://www.genome.jp/kegg/, accessed on 14 April 2014). Data were analyzed using the Genesifter Lab Edition, V2, (Geospiza Inc., Seattle, WA, USA) to show genes significantly affected by curcumin. All RefSeq and UniProt references were taken from genecards.org.

## 3. Results

### 3.1. Growth Inhibitory Activity of Phytochemicals on Cervical Cancer Cells

**Figure 1:**
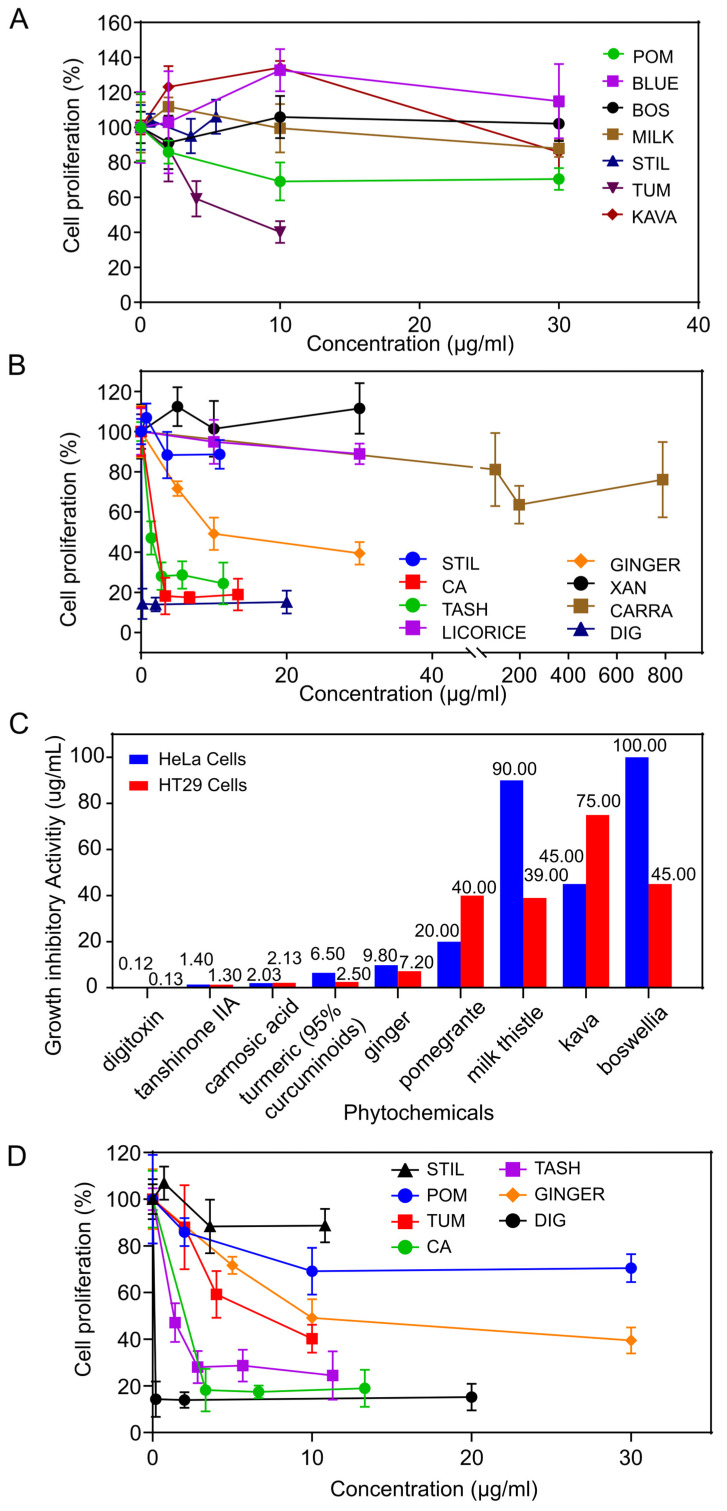
Growth inhibitory activity of herbal medicines on HeLa cervical and HT29 colon cancer cells. Cells were exposed to increasing concentrations of agents for 96 h and the number of viable cells determined by the MTT assay. (**A**) Set 1: POM, pomegranate; BLUE, blueberry; BOS, Boswellia; MILK, milk thistle; STIL, stilbene; TUM, turmeric; KAVA, kava; (**B**) Set 2: STIL, stilbene; CA, carnosic acid; TASH, tanshinone IIA; LICORICE, licorice; GINGER, ginger; XAN, xanthine; CARRA, carrageenan; DIG, digoxin; (**C**) Growth inhibitory activity of phytochemicals on HeLa vs HT29 cells; (**D**) Growth inhibitory activity of the phytochemicals with the highest activity, on HeLa cells. STIL, stilbene; POM, pomegranate; TUM, turmeric; CA, carnosic acid; TASH, tanshinone IIA; GINGER, ginger, DIG, digoxin.

The phytochemicals were first tested on HeLa cervical cancer cells to determine their growth inhibitory effects. These results are displayed in Figure 1A,B, with summaries of the IC_50_ values, i.e., the concentration of the most active phytochemicals that caused 50% cell proliferation inhibition of HeLa cells, as shown in Table 1A.

In our first set of experiments, turmeric (95% curcuminoids) exhibited the highest activity: 6.5 μg/mL; followed by pomegranate: 20 μg/mL; kava: 45 μg/mL; and milk thistle: 90 μg/mL (Figure 1A); it is important that turmeric displayed significant activity at concentrations as low as 2 μg/mL (*p* < 0.0001). The rest of the phytochemicals (stilbene, blueberry, carrageenan) had IC_50_s greater than 100 μg/mL, and are considered to have weak activity.

In the second set of experiments, among the compounds, digoxin had the highest activity of 0.12 μg/mL (0.15 μM), followed by: tanshinone IIA: 1.33 μg/mL (4.52 μM); carnosic acid: 2.03 μg/mL (6.11 μM). The ginger extract displayed intermediate activity; 9.8 μg/mL (Figure 1B); while licorice extract and carrageenan (IC_50_ values > 100 ug/mL or 250 μM, respectively) had little activity, as shown in Figure 1B.

In these experiments, we also examined the effect of all the compounds and extracts on HT29 colon cancer cells to assess specificity. In some instances, herbs exhibited greater activity on particular cell lines. Turmeric (95% curcuminoids), milk thistle (80% silymarin) and Boswellia (68% boswellic acids) were more active on HT29 compared to HeLa cells; whereas pomegranate (40% ellagic acid) and kava (30% kavalactones) were more active on HeLa versus HT29 cells (Table 1A, Figure 1C).

In summary, the relative order of activity (decreasing) of the phytochemicals on HeLa and HT29 cells is as follows: HeLa: (compounds) digoxin, tanshinone IIA, carnosic acid; (extracts) turmeric, ginger, pomegranate, kava, milk thistle, Boswellia; HT29: (compounds) tanshinone IIA, carnosic acid; (extracts) turmeric, ginger, milk thistle, pomegranate, Boswellia, kava.

Figure 1D displays the growth inhibitory activity of the phytochemicals with the highest activity, on HeLa cells; the most active are pure compounds.

### 3.2. Growth Inhibitory Effects on W12 Cervical Cells

To further explore the action of these phytochemicals, we assayed the effects of the most active phytochemicals on W12 cervical cells, which contain the HPV16 genome in different states of integration (five W12 cell lines containing episomal or integrated DNA), as well as 3T3 feeder cells. Table 1B summarizes the growth inhibitory activity of the most active phytochemicals on W12 cells.

**Figure 2:**
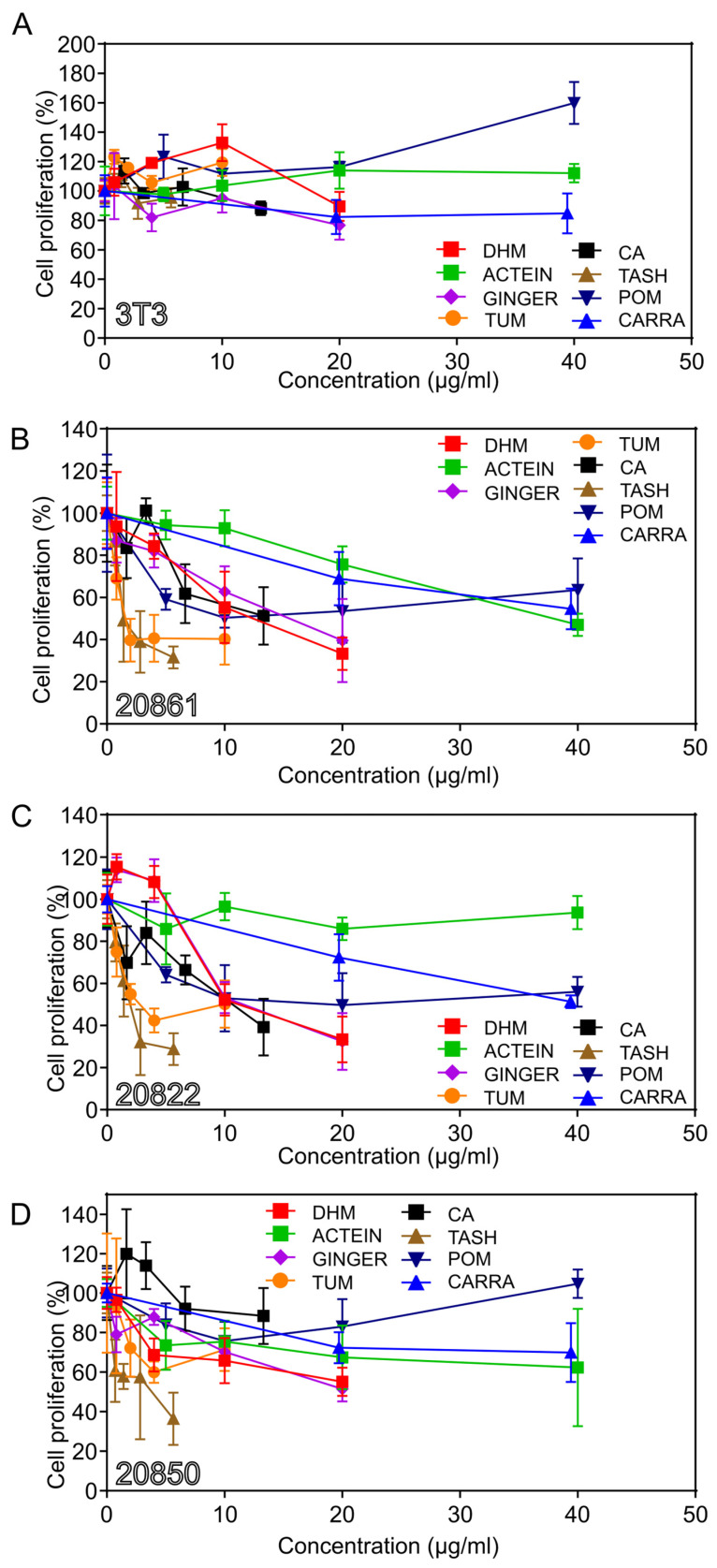
Growth inhibitory activity of herbal medicines on W12 cervical and 3T3 feeder cells. (**A**–**D**), (**A**) 3T3; (**B**) 20861; (**C**) 20822; and (**D**) 20850 cells; Cells were exposed to increasing concentrations of agents for 120 h and the number of viable cells determined by the MTT assay. Herbal components were added directly to the cells in the 96-well plates. DHM, dihydromethysticin; ACTEIN, actein; GINGER, ginger; TUM, turmeric; CA, carnosic acid; TASH, tanshinone IIA; POME, pomegranate; CARRA, carrageenan.

The feeder 3T3 cells were relatively resistant to the growth inhibitory activity of the phytochemicals (Figure 2A). Since the feeder cells were present in relatively low numbers and they are relatively resistant to the phytochemicals, the feeder cells do not appear to have a significant effect on the results.

The relative order of activity of the phytochemicals on W12 cells is similar to that on HeLa cells. For type 2 integrant (20861) cells, the descending order of activity (Figure 2B) is: (IC_50_ values in parentheses): (compounds) tanshinone IIA (1.4 μg/mL; 4.8 μM); carnosic acid (14.1 μg/mL; 42.4 μM); DHM (12.4 μg/mL; 44.9 μM); and actein (37.9 μg/mL; 56.0 μM); (fractions) turmeric; (1.6 μg/mL); pomegranate (10 μg/mL); and ginger (15.5 μg/mL) (Figure 2B).

The three W12 cell lines displayed similar patterns of activity. However, there were notable differences: ginger was highly active on 20822 cells; and carrageenan (after sonication), on 20850 cells; in a repeat experiment, carrageenan exhibited the inverse pattern of activity.

We also assayed the activity on type 2 20862 cells; the order of activity was similar to that on type 2 20861 cells; however, carnosic acid was more active on 20862 compared to 20861 cells. Turmeric was the most active fraction (1.96 μg/mL).

Turmeric and tanshinone IIA showed significant activity on 20850 (episomal) cells at low concentrations: turmeric 10 μg/mL; (*p* = 0.0402) and tanshinone IIA 2.5 μM; (*p* = 0.0058).

**Figure 3:**
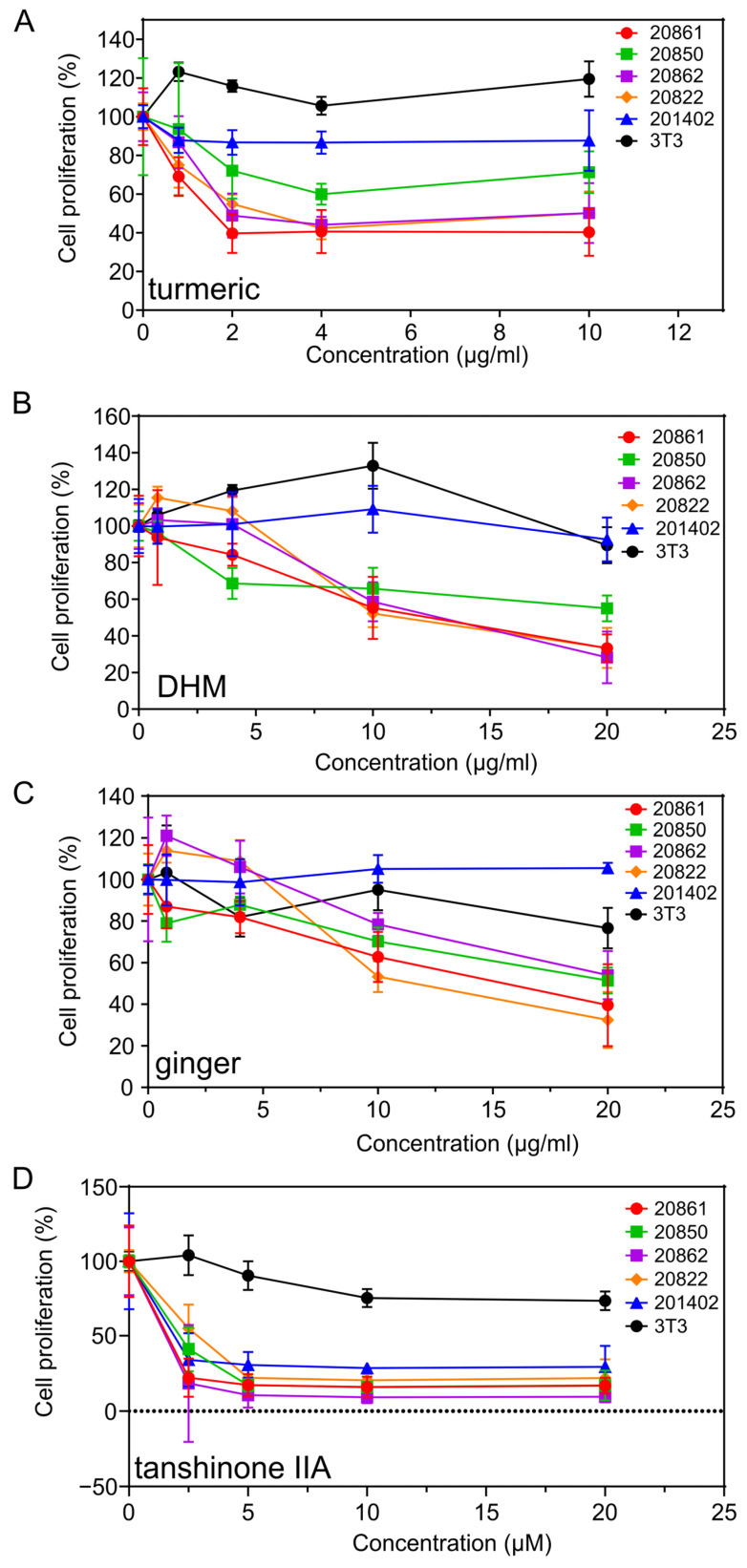
Growth inhibitory activity of herbal medicines on W12 cervical and 3T3 feeder cells; (**A**–**D**) W12 cervical precancer cells; (**A**) turmeric, (**B**) DHM, (**C**) ginger, (**D**) tanshinone IIA; Cells were exposed to increasing concentrations of agents for 120 h and the number of viable cells determined by the MTT assay.

The relative sensitivity of the W12 cell lines to some of the most active phytochemicals, including turmeric, DHM, ginger, and tanshinone IIA, is illustrated in Figure 3A–D. The activity of digoxin was also tested; the order of decreasing activity was: 20861 (0.0037 μg/mL), 20822 (0.008 μg/mL), 20862 (0.0245 μg/mL), 20850 (>0.025 μg/mL), and 3T3 (>10 μg/mL) (Einbond “Synergistic combinations of digoxin” in preparation [20]). Digoxin and tanshinone IIA exhibit potent activity against the growth of W12 cells. Since digoxin has a narrow therapeutic index, it was not included in further studies.

In summary, the approximate order of decreasing activity of the phytochemicals on W12 cells is as follows: (compounds) digoxin, tanshinone IIA, carnosic acid, actein or DHM; carrageenan (fractions) turmeric, ginger.

Regarding the W12 cells, the approximate order of decreasing sensitivity is: 20861, 20862, 20822, and 20850 (Figure 3A–D). In the majority of experiments the relative rate of growth was 20861 ≥ 20862 > 20822 > 20850. The relative sensitivity may relate to the growth rate of the cells and/or to the extent of transformation.

### 3.3. The Effects of Combinations of Phytochemicals

**Figure 4:**
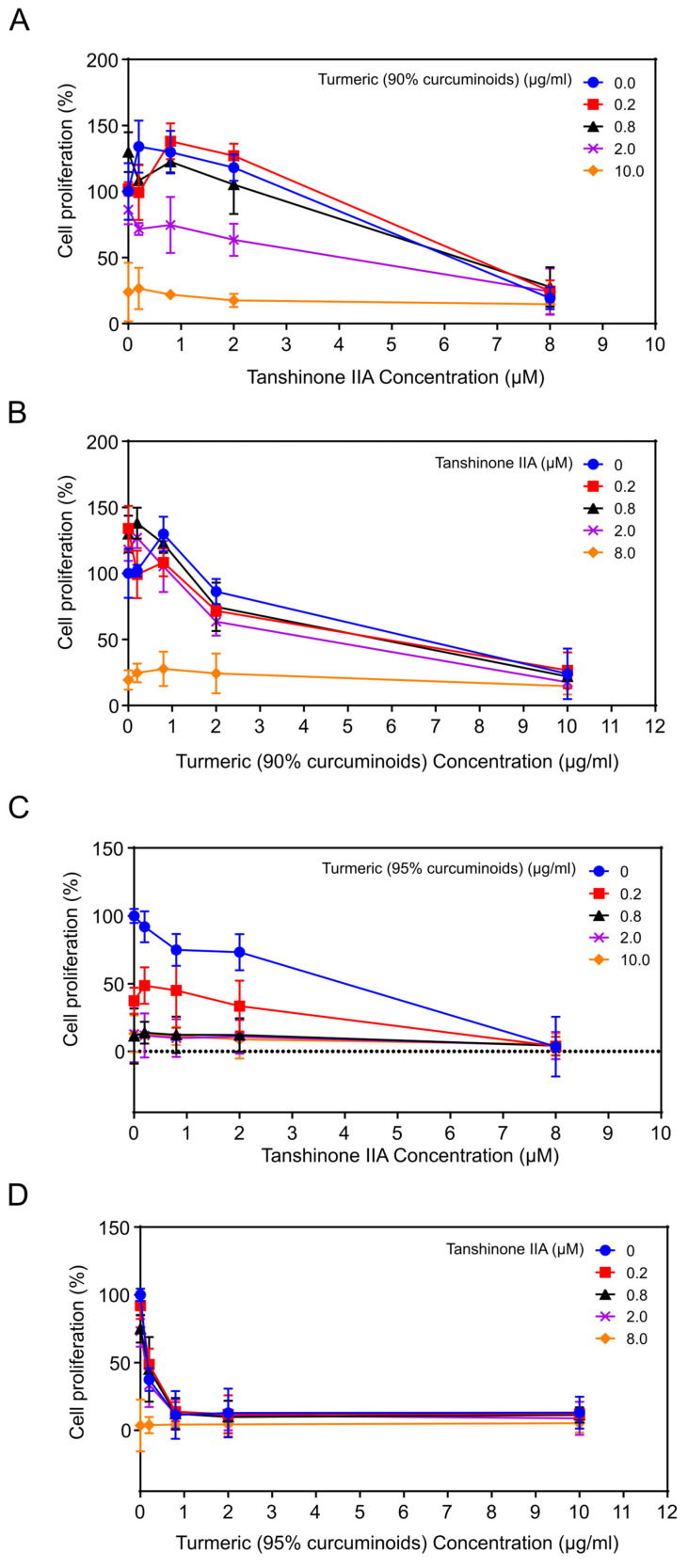
Growth inhibitory activity of combinations of herbal components: tanshinone IIA and turmeric (90 or 95% curcuminoids) on HeLa cells. (**A**,**B**) Combination of tanshinone IIA plus turmeric (90% curcuminoids); (**A**) *x*-axis: tanshinone IIA; (**B**) *x*-axis; turmeric (90% curcuminoids); (**C**,**D**) Combination of tanshinone IIA plus turmeric (95% curcuminoids); (**C**) *x*-axis: tanshinone IIA; (**D**) *x*-axis; turmeric (95% curcuminoids). We treated HeLa cells with all combinations of 4 concentrations of each of the agents tested and a solvent control [19]. Cells were exposed to increasing concentrations of agents for 96 h and the number of viable cells determined by the EZQUANT assay.

To determine optimal therapeutic candidates to prevent and treat cervical cancer, we assayed the growth inhibitory effects of combinations of the most active, relatively nontoxic, phytochemicals on HeLa cervical cancer cells. We selected phytochemicals that potentially target various pathways utilized by HPV to replicate and induce viral transformation. The combinations tested were as follows: tanshinone IIA plus turmeric (90 or 95% curcuminoids) (Figure 4A–D); or turmeric (95% curcuminoids) plus ginger or carrageenan (Sections S1.1 and S1.2; Figure S2A–D). To assess possible synergistic effects, cells were treated with all combinations of 4 concentrations of each of the agents tested and a solvent control [19].

#### 3.3.1. Combination of Tanshinone IIA Plus Turmeric (90% Curcuminoids)

The IC_50_ values for tanshinone IIA and turmeric (90% curcuminoids) alone were approximately 6 μM and 6.25 μg/mL, respectively (Figure 4A,B). Increasing concentrations of curcumin were combined with increasing concentrations of tanshinone IIA (Figure 4A,B). At a dose of tanshinone IIA 0.2 μM, the percentage of viable cells decreased from 134.02% with tanshinone IIA alone to 99.23% with curcumin 0.2 μg/mL, (increased) to 108.26% with curcumin 0.8 μg/mL, to 71.65% with curcumin 2 μg/mL, and to 26.54% with curcumin 10 μg/mL (*p* < 0.01). Thus, curcumin enhances the growth inhibitory effect of tanshinone IIA in the human cervical cancer cell line HeLa. The percent survival after treatment with tanshinone IIA (0.2 μM) and turmeric (90% curcuminoids) (2 μM) alone or combined is as follows: tanshinone IIA (0.2 μM), 134%; turmeric (90% curcuminoids) (2 μΜ), 86.3%; combination, 71.7%.

To quantify the interaction between the compounds, we calculated the combination index, using the method of Chou and Talalay [19]. The Combination Index (CI) for the combination of tanshinone IIA 2 μΜ and curcumin 8 μg/mL was approximately 0.67; while the CI for the combination of tanshinone IIA 10 μΜ and curcumin 2 μg/mL was approximately 0.64, both indicating moderate synergy.

#### 3.3.2. Combination of Tanshinone IIA Plus Turmeric (95% Curcuminoids)

The IC_50_ values for tanshinone IIA and turmeric (95% curcuminoids) alone were approximately 4 µM and 0.16 μg/mL, respectively, on HeLa cervical cancer cells.

When increasing concentrations of both tanshinone IIA and turmeric (95% curcuminoids) were combined (Figure 4C,D; Statistical analysis: Table S3), at a dose of tanshinone IIA 0.2 µΜ, the percent viable cells decreased from 91.96% with tanshinone IIA alone, to 48.65% with curcumin 0.2 μg/mL, to 13.76% with curcumin 0.8 μg/mL, to 11.75% with curcumin 2.0 μg/mL, and to 12.16% with curcumin 10 μg/mL (*p* < 0.01) (Table S3). Thus, curcumin enhances the growth inhibitory effect of tanshinone IIA on the human cervical cancer cell line HeLa.

The CI for the combination of tanshinone IIA 0.2 µΜ and turmeric (95% curcuminoids) 0.2 µg/mL was <0.6, indicating strong synergy. The final percent cell viability was very low suggesting the combination may be effective to treat cancer. The combination of turmeric (95% curcuminoids) and tanshinone IIA was more effective compared to the combination of turmeric (90%) curcuminoids and tanshinone IIA.

#### 3.3.3. Combination of Turmeric (95% Curcuminoids) Plus Ginger or Carrageenan

Turmeric plus ginger: In preliminary experiments the IC_50_ values for turmeric (95% curcuminoids) and ginger alone were approximately 5.6 μg/mL and 19 μg/mL, respectively, on HeLa cervical cancer cells. Curcumin enhances the growth inhibitory effect of ginger on HeLa cells (Section S1.1; Figure S2A,B). The CI for the combination of turmeric (95% curcuminoids) 2 μg/mL and ginger 10 μg/mL was approximately 0.53, indicating strong synergy.

Turmeric plus carrageenan: Since carrageenan interferes with HPV16 attachment to the cell and later steps of viral replication [16], we examined the growth inhibitory effects of combinations of carrageenan and turmeric (95% curcuminoids) on HeLa cells. In preliminary experiments, the IC_50_ values for turmeric (95% curcuminoids) and carrageenan alone (after sonication) were approximately 0.4 μg/mL and 2.0 μM, respectively, on HeLa cervical cancer cells (Section S1.2; Figure S2C,D). Sonication may degrade the structure of carrageenan [15,17]. We will study this in future experiments. Curcumin enhances the growth inhibitory effect of carrageenan on the human cervical cancer cell line HeLa (Section S1.2; Figure S2C,D). The CI for the combination of turmeric (95% curcuminoids) 0.1 ug/mL and carrageenan 2 μg/mL was approximately 0.78, indicating moderate synergy.

### 3.4. The Effects of Tanshinone IIA on the Expression of p53 and HPV16 mRNAs

**Figure 5:**
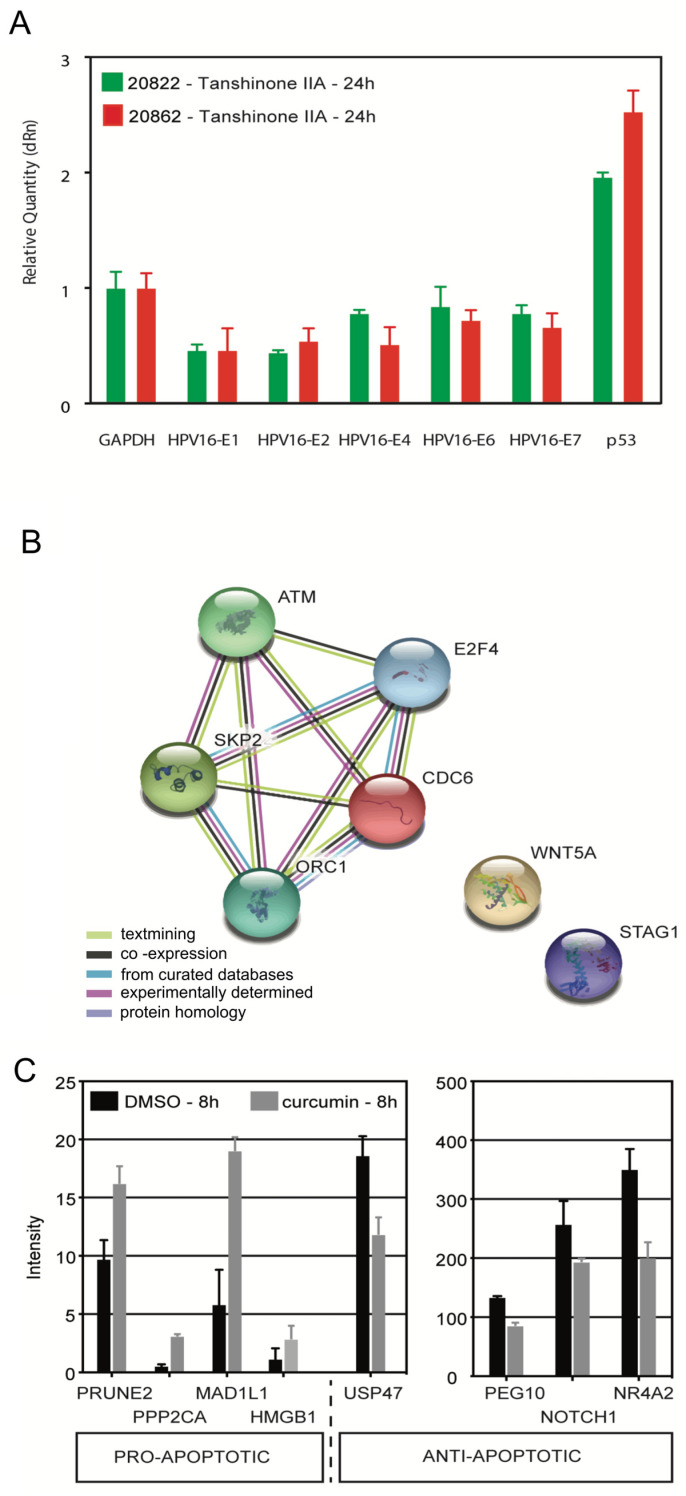
Real-time RT-PCR analysis of RNA obtained after treating W12 cells with tanshinone IIA; Gene expression analysis of the effects of curcumin on HeLa cells, at 8 h. (**A**) Real-time RT-PCR: Effect of tanshinone IIA on p53 and HPV16 mRNAs: E1, E2, E4, E6 and E7 in 20822 and 20862 cells, at 24 h; (**A**) W12 were treated with tanshinone IIA (5 μM); extracts were prepared and analyzed by Real-time RT-PCR, as described in Materials and Methods. Fold change indicates relative expression in tanshinone IIA treated versus control treated cells. (**B**,**C**) Gene expression analysis of the effects of curcumin on HeLa cells at 8 h; (**B**) STRING (Functional Protein Association Networks: Search Tool for Retrieving Interacting Genes/proteins) analysis of the effects of curcumin on HeLa cells; (**C**) Pro- and Anti-apoptotic genes significantly altered by curcumin (*p* < 0.05, except NOTCH1): Curcumin vs. vehicle (*p* value): Pro-Apoptotic genes; CD30 (0.0059); HMGB1 (0.0469); Prosome (0.0026); Anti-Apoptotic genes; ROCK (0.0018); PEG10 (<0.0001); NOTCH1 (0.0307). HeLa cells were treated with curcumin (32 μM) and collected for RNA extraction at 8 h. Samples were prepared for gene expression analysis, as described in Section 2.7.

To investigate the mode of action of tanshinone IIA, we performed RT PCR for p53 and HPV16 mRNAs on W12 type 1 (20822) and type 2 (20862) integrant cells. The results are displayed in Figure 5A and Table S2 and (Statistical analysis) Table S4.

#### 3.4.1. Effect of Tanshinone IIA on the Expression of p53

The sensitive tool RT-PCR showed that tanshinone IIA activated the expression of p53 at 24 h in the two W12 cell types tested, type 1 and 2 integrants (relative fold: 20822: 1.96; 20862: 2.53). The difference between the relative levels of p53 mRNAs in the 2 cell types (20822 vs. 20862) at 24 h is significant (*p* < 0.01) (Table S2). The activation was greater on type 2 compared to type 1 integrant cells, which may relate to the state of integration of the HPV16 genome.

To explore an effect on p53 protein levels, it is of interest to monitor the effect of tanshinone IIA on the mRNA levels of genes responsive to p53, such as p21 (Cyclin Dependent Kinase Inhibitor 1A) and MDM2 (MDM2 Proto-Oncogene). MDM2, which encodes a nuclear-localized E3 ubiquitin ligase, is the major negative regulator of p53. In preliminary experiments on W12 20822 (type 1 integrant) cells tanshinone IIA activated the expression of p21 (1.41-fold) and repressed the expression of MDM2 (0.78-fold), at 24 h. The effect on MDM2 is the inverse of the effect on p53.

#### 3.4.2. Effect of Tanshinone IIA on the Expression of HPV16 mRNAs

Concerning HPV16 mRNAs, tanshinone IIA repressed the expression of HPV16 mRNAs (E1, E2, E4, E6, E7) in both W12 cell types tested (type 1 and 2 integrants). The relative fold reduction is shown in Figure 5A and Table S2. The early genes were more sensitive than the late gene E4. The reduction in expression of the early gene E2 was greater in 20822 (0.44) vs. 20862 (0.54) cells; while the reduction in expression of the late genes (E4, E6, E7) was greater in 20862 versus 20822 cells. The difference between the relative levels of HPV16 mRNAs in the 2 cell types, 20822 vs. 20862, at 24 h, is significant for E4 (*p* < 0.01) (Tables S2 and (Statistical analysis) S4).

The two cell types (types 1 and 2 integrants) were about equally sensitive by RT-PCR assay. This corresponds to the relative growth inhibitory effect (IC_50_ values in parentheses): 20822 (2.1 μM); 20862 (2.8 μM).

### 3.5. Gene Expression Analysis of the Effects of Curcumin

To better understand the mode of action of curcumin, gene expression analysis was performed on HeLa cervical cancer cells. Curcumin (32 mg/mL) at 8 h, activates the expression of cell cycle control genes (*p* < 0.05) (Table 2). Key genes are ATM (36-fold), CDC6, ORC1L, E2F4, SKP2 and STAG1 (*p* < 0.01, fold > 2). These genes mediate the DNA damage response, cell cycle progression and replication initiation. The functions of these genes are as follows: ATM: Serine/threonine protein kinase, acts as a DNA damage sensor; after double-strand breaks, it activates checkpoint signaling and stabilizes p53 [21]; CDC6 (Cell division control protein 6 homolog) functions in checkpoint control, oversees DNA replication; ORC1 (Origin recognition complex subunit; pre-replication complex) is essential to initiate DNA replication; E2F4 (Transcription factor E2F4) acts in control of G1 to S cell cycle progression; SKP2 (S-phase kinase-associated protein 2) component ubiquitin ligase complex, ubiquitinates and degrades target proteins involved in cell cycle progression, signal transduction and transcription; and functions in regulation of G1/S transition; STAG1 (Cohesion subunit SA-1) functions in spindle pole assembly in mitosis; GSK3 (glycogen synthase kinase 3) is involved in cell migration.

STRING (Functional Protein Association Networks: Search Tool for Retrieving Interacting Genes/proteins) analysis was used to reveal interconnections among the cell cycle proteins, as displayed in Figure 5B. These interconnected genes are involved in checkpoint control (ATM; CDC6), DNA replication (ORC1L), and cell cycle control (E2F4, SKP2, and STAG1). These genes interact with p53. In addition, curcumin activated the expression of apoptotic genes (Prune2, PPP2CA, and HMGB1), and the cell cycle gene MAD1L1, and repressed the expression of anti-apoptotic genes (PEG10, NOTCH1, IL10, USP47, and NR4A2), as displayed in Figure 5C (*p* < 0.05, except NOTCH1 *p* = 0.0518 and HMGB1 *p* = 0.1154; (Statistical analysis) Table S5 [12].

Further, curcumin downregulated the gene expression of factors secreted by tumor cells known to induce immunosuppression (VEGFA, PDGF, IL4, OSM, CXCR4 and Fas ligand). and activated the expression of factors secreted by tumor cells, which cause immunostimulation, IL2.

### 3.6. Effect of Curcumin on the Activity of the Na^+^/K^+^-ATPase 

Since the Na^+^/K^+^-ATPase plays a role in the development and progression of cervical cancer, we assayed the effect of curcumin on the activity of the enzyme. Curcumin inhibited the activity of the enzyme; the IC_50_ of curcumin was 2 μM compared to <0.1 for digitoxin, as previously described [22].

### 3.7. Binding (Molecular Docking) of Drugs to Na^+^/K^+^-ATPase 

**Figure 6:**
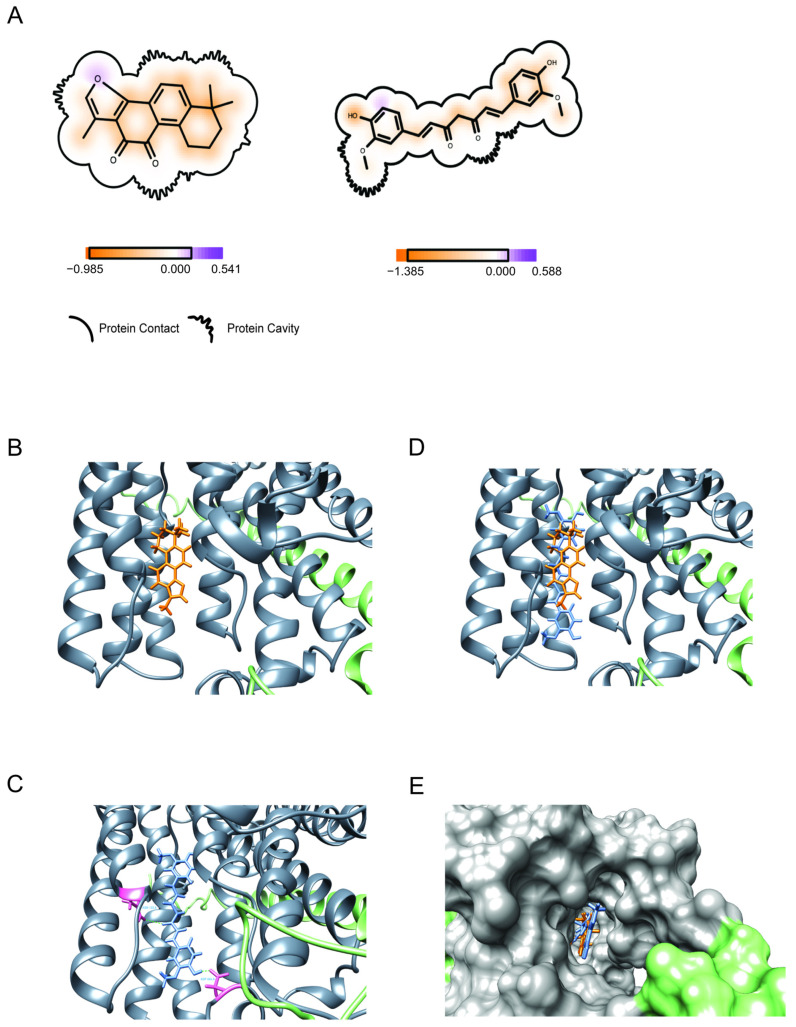
Molecular docking of curcumin and tanshinone IIA, alone and in combination, to the Na^+^/K^+^-ATPase. The molecular docking of tanshinone IIA to Na^+^/K^+^-ATPase uses the OMEGA of OpenEye toolkit, (**A**) Structures: tanshinone IIA and curcumin; (**B**) Tanshinone IIA binds to the Na^+^/K^+^-ATPase; FRED docking result of tanshinone IIA (orange) to Na^+^/K^+^-ATPase (PDB id: 7DDI (A chain in gray, B chain in light green). (**C**) Curcumin binds to Na^+^/K^+^-ATPase ; FRED docking result of curcumin to Na^+^/K^+^-ATPase (PDB id: 7DDI (A chain in gray, B chain in light green). The ligand curcumin (blue) forms hydrogen bonds (green dashed line) with the residues ASN 122 and ASP 884 of the protein chain A (residue structures highlighted in pink). (**D**) The combination of tanshinone IIA and curcumin binds to Na^+^/K^+^-ATPase; (**E**) The combination of tanshinone IIA and curcumin binds to Na^+^/K^+^-ATPase with solvation; Figure: FRED docking results of curcumin (blue) and tanshinone IIA (orange) to Na^+^/K^+^-ATPase (PDB id: 7DDI. The docking is performed separately for each ligand with protein. The best poses of two ligands aligned in the Na^+^/K^+^-ATPase active site and protein is shown as its secondary structure (the two ligands aligned in the protein pocket). The protein is present with the solvent-accessible surface, which is generated by Chimera in default parameters (Figure S3).

To gain insight into the mode of action of tanshinone IIA and curcumin (Structures, Figure 6A,B and Figure S1A,B), alone and in combination, we assessed their binding to the Na^+^/K^+^-ATPase, using molecular docking.

#### 3.7.1. Binding of Tanshinone IIA

For tanshinone IIA, the docking score was −10.45. The majority of the score comes from the shape evaluation (highlighted in the red box) (Figure 6B and Figure S3A), which means the ligand shape is fit to the protein active site (docking pocket). The hydrogen bond score and the residue fingerprint show no H-bonds formed between the protein and tanshinone IIA.

#### 3.7.2. Binding of Curcumin

Curcumin binds with high affinity; the docking score was −12.74, which is comparable to the reported minimal binding score of digitoxin (−12.5 kcal/mol) and exceeds its reported average score (−11.8 kcal/mol) [23]. As shown in Figure 6C and Figure S3B, the ligand (curcumin) (blue) forms hydrogen bonds (green dashed line) with the residues ASN 122 and ASP 884 of protein chain A (residue structures highlighted in pink).

#### 3.7.3. Binding of the Combination of Tanshinone IIA and Curcumin

To better understand the synergistic effects of tanshinone IIA and curcumin, we assayed the binding of the combination to the Na^+^/K^+^-ATPase. The best poses of the two ligands aligned in the Na^+^/K^+^-ATPase active site and protein are shown as the secondary structure in Figure 6D; the two ligands aligned in the protein pocket with different patterns. The protein is present with the solvent-accessible surface, which is generated by Chimera in default parameters.

This figure shows that tanshinone IIA fits in the deep place of the protein pocket.

The tanshinone IIA docked position is well aligned with that of curcumin. This shows that curcumin and tanshinone IIA possibly interact with the protein at similar positions.

However, tanshinone IA did not share similar protein- ligand interactions (H-bonds) with curcumin to the Na^+^/K^+^-ATPase. Figure 6E displays the binding of tanshinone IIA and curcumin to the Na^+^/K^+^-ATPase with solvation.

## 4. Discussion

This study explores the effects of plant compounds on W12 cervical precancer cells and bioelectric signaling. The results show that a variety of individual phytochemicals: tanshinone IIA, digoxin, carnosic acid, DHM, carrageenan and actein; as well as enriched extracts of turmeric, pomegranate and ginger inhibit the growth of W12 cells. These cells are derived from a cervical precancerous lesion, and harbor episomal or integrated DNA. It is especially important that these phytochemicals inhibit the growth of W12E episomal 20850 cells, containing episomal HPV16. This highlights their potential to treat HPV infections and CIN1 precancerous lesions, which also contain episomal HPV, in women at risk of progression to cancer.

In addition, DHM, actein and kava inhibit the growth of HPV18 positive HeLa cells. Our results align with previous research showing the inhibitory effects of tanshinone IIA [6,7,8], digoxin [24], carnosic acid [25], curcumin [26], ginger [27], and pomegranate [28], and not blueberry [29], on HeLa cells,

Bioelectric signaling is linked to cancer growth and inhibition via processes such as ATPase regulated apoptosis [30], and the MAPK signaling cascade [31]. Ion channel molecules and gap junctions create electrical gradients (Vmem) that maintain the balance between normal patterns of cell growth and the development of cancer [32]. Curcumin and tanshinone IIA were the most potent, relatively nontoxic, single agents; both strongly inhibit the growth of W12 cells. We examined their ability to bind to the ion channel molecule, the Na^+^/K^+^-ATPase, using molecular docking. Curcumin potentially binds with high affinity, comparable to that of digitoxin, while tanshinone IIA shows weaker binding affinity. To our knowledge, this is the first report showing that curcumin and tanshinone IIA may bind to the Na^+^/K^+^-ATPase.

Pertaining to the W12 cells, some herbal compounds are more active on different clones of W12 cells. The approximate order of decreasing activity for tanshinone IIA, DHM and turmeric is: (type 2) 20861, (type 1) 20822, and (episomal) 20850. This variance could relate to the growth rate of the cells and/or to intrinsic genetic alterations associated with transformation. For most experiments, the sensitivity to compounds paralleled the relative rate of growth, which was in decreasing order: (type 2) 20861, 20862; (type 1) 20822; (episomal) 20850. Interestingly, for carrageenan (after sonication), preliminary experiments indicate the order may be the reverse: episomal > type 1 > type 2 clones, but this requires further investigation.

Previous studies have shown that curcumin preferentially inhibits the growth of cervical cancer cell lines, while sparing nonmalignant cells [33], indicating that this compound possesses specificity and may have limited toxicity, *in vivo*. Additionally, curcumin formulated as VaCurin, an oil-in-water emulsion, showed no adverse effects on body weight or the vaginal epithelium, when applied intravaginally. Our combination studies suggest it may be beneficial to combine carrageenan with these creams to prevent the transmission of HPV viruses. Topical application will allow significant tissue concentration and lessen systemic side effects.

Pan *et al*. (2010, 2013) have shown that tanshinone IIA suppresses the expression of E6 and E7 and induces the expression of p53 and pRB, which alter proteins that mediate proliferation, the cell cycle and apoptosis [6,8]. In addition, Mungala *et al*. (2015) [8] observed a decrease in the levels of E6 and E7 oncoproteins after treatment with tanshinone IIA. E6 and E7 have been shown to induce degradation of p53. Consistent with this, Mungala *et al*. (2015) reported an increase in the levels of p53 protein after treating HeLa cells with 0–10 μM tanshinone IIA for 24 h. Tanshinone IIA induces apoptosis through mitochondrial intrinsic and ER stress pathways [7] and altering microtubule assembly [6].

To better understand the action of tanshinone IIA, we examined the effect of tanshinone IIA on W12 cells, using RT-PCR. The present studies show that tanshinone IIA activated the expression of p53 in W12 integrant clones. The relative order of activity was type 2 > type 1 integrant, which may relate to the extent of transformation. In the future, we will examine the effect of tanshinone IIA on the protein levels of p53 and activated p53, in W12 cells.

Further, tanshinone IIA suppresses the expression of HPV16 early genes, E1, E2 and E4, in addition to E6 and E7, in W12 type 1 and 2 integrants. It is important to highlight that tanshinone IIA repressed the expression of E1 and E2. These genes are required for viral replication; yet it has been difficult to find agents to target them [34].

To gain insight into the action of curcumin, we examined the effect of curcumin on HeLa cells, using gene expression analysis. Curcumin activated the expression of genes that mediate checkpoint control (ATM; CDC6), DNA replication (ORC1), and cell cycle control (E2F4, SKP2); these genes interact with p53; ATM stabilizes p53 after DNA damage [21]. In addition, curcumin activated the expression of apoptotic genes and repressed the expression of anti-apoptotic genes [12]. Further, curcumin downregulated the expression of factors secreted by tumor cells known to cause immunosuppression and activated antitumor mediators of inflammation.

In this study we showed that tanshinone IIA synergizes with curcumin in cervical cancer cells. The combination will permit the use of lower doses of the agents, which will lessen the side effects, and reduce the development of resistance. To explain the synergy, agents may affect similar or distinct pathways, or one may potentiate or stabilize the effects of the second.

Tanshinone IIA and curcumin affect the oncogene P53, which is crucial for DNA repair and apoptosis. Our findings indicate tanshinone IIA activates the expression of p53 in W12 integrant cells. Curcumin activates the expression of genes that function in checkpoint control (ATM: 36-fold), DNA replication and cell cycle control; these genes interact with p53; ATM (Serine/threonine protein kinase) stabilizes p53 after DNA damage [21]. In previous experiments, we have shown that curcumin increases the level of p53 and activated p53, acetyl p53, proteins, in TC-1 lung epithelial cells [12].

Cardiotonic steroids (CS) do not affect H1299 (lung cancer) cells, which lack p53, indicating that the effects of cardiotonic steroids depend on the presence of p53 [35]. CSs impact the Na^+^/K^+^-ATPase, a p-type transmembrane protein, which plays a crucial role in intracellular bioelectric signaling, and the development and progression of cancer. In cervical and other cancer cells, it regulates: ionic balance [36], the Src family kinases [22], cell migration, motility, and invasion [37]; suppresses proinflammatory signaling [38]; and reprograms the immune environment [39]. The anticancer effects of cardiac steroids (CS) depend on their concentration. At concentrations above the IC_50_, CSs inhibit the Na^+^/K^+^-ATPase, disrupt ion homeostasis, and induce apoptosis. At concentrations well below the IC_50_, they activate signaling pathways, depending on the cell type, including p21 retention in the cell cycle, SrcK activation or inhibition, VRAC regulation of cell volume, and inhibition of cell motility and angiogenesis [40]. The Na^+^/K^+^-ATPase and voltage-gated sodium channels (VGSCs) are upregulated in most malignant carcinomas [41]. In future studies, we will assay the level of the Na^+^/K^+^-ATPase in W12 precancer cells.

We hypothesize that tanshinone IIA and curcumin, alone or in combination, may modulate Na^+^/K^+^-ATPase function and influence bioelectric signaling and cancer progression in cervical cancer cells. Tanshinone IIA is currently used in China to treat heart disorders [42]. It has a range of effects: comprising cardioprotective, anti-inflammatory, antioxidant, and antitumor properties. Studies indicate tanshinone IIA protects cardiomyocytes and guards against heart failure after myocardial infarction, by activating the AMPKs/mTOR-dependent autophagy pathway [43].

Tanshinone IIA and curcumin appear to have different effects on the activity of the Na^+^/K^+^-ATPase . Tanshinone IIA increases levels of the Na^+^/K^+^-ATPase in adult Sprague–Dawley rats [44]. Our results indicate curcumin inhibits the activity of the enzyme. While Singh *et al*. (2015) [45] reported that curcumin inhibits the activity at higher concentrations and upregulates the activity at lower concentrations and *in vivo*.

We explored interactions between curcumin and tanshinone IIA with the Na^+^/K^+^-ATPase to gain insights into their potential modes of action in treating cervical cancer. Molecular docking analysis revealed that tanshinone IIA and curcumin bind to the Na^+^/K^+^-ATPase with docking scores of −10.45 or −12.74, respectively. The binding of curcumin is strong, comparable to that of digitoxin, whereas tanshinone IIA shows a weaker binding affinity.

Regarding tanshinone IIA, the main part of the score comes from the shape evaluation, which means the ligand shape is fit to the protein active site (docking pocket). The hydrogen bond score and the residue fingerprint show there are no H-bonds formed between the protein and tanshinone IIA. The reason for this may be that tanshinone IIA: (1) is too small for the active site of the protein, and cannot form a stable interaction with the protein, and (2) has few H-bond acceptors or donors (only three H-acceptors)

In contrast, curcumin formed hydrogen bonds with ASN 122 and ASP 884 of the Na^+^/K^+^-ATPase protein chain. This difference suggests a distinct mode of action for curcumin compared to tanshinone IIA. Combined docking indicates tanshinone IIA and curcumin may interact with the Na^+^/K^+^-ATPase at similar positions. However, tanshinone IIA did not share similar protein- ligand interactions (H-bonds) with curcumin to the Na^+^/K^+^-ATPase. The possibility that tanshinone IIA might alter the binding dynamics of curcumin warrants further study.

It is important to be cautious when interpreting the results, since the active site or binding site is manually defined at the beginning of the docking process. Further studies, such as competition assays or binding studies, will be needed to confirm our findings and to clarify the effects of tanshinone IIA on the binding of curcumin.

In addition to preclinical studies, a proof-of-concept clinical case report targeted the Na^+^/K^+^-ATPase , using targeted osmotic lysis (TOL) for the treatment of late-stage cervical carcinoma [41]. This innovative approach, which involves the pharmacological blockade of the Na^+^/K^+^-ATPase, was used to treat a patient with advanced stage IIB squamous cell carcinoma of the cervix. The technology, which relies on the simultaneous stimulation of VGSCs and inhibition of Na^+^/K^+^-ATPase with a cardiac glycoside, results in rapid and selective osmotic lysis of carcinoma cells. This clinical case emphasizes the potential of Na^+^/K^+^-ATPase -targeted therapies.

Cancer may be viewed as a defect in cellular organization, resulting from the loss of communication of bioelectric pathways; these pathways enable cells to form networks, instead of remaining unicellular [46]. The microenvironment of the tumor is bioelectric, as well as chemical, and may exert effects across long distances [47]. Vmem, produced by ion channels and gap junctions, is a critical property of the microenvironment that controls the balance between normal patterns of cell growth and the onset of carcinogenesis [32]. Understanding the roles of bioelectricity in forming patterns may allow the activation of pathways to normalize tumor tissue [48,49]. Compounds, such as curcumin and tanshinone IIA, which interact with the Na^+^/K^+^-ATPase, may have a significant influence on the development and progression of cancer. Lobikin *et al*. [32] report that the proliferation of certain tumor cells depends on voltage gated potassium channels, such as the K^+^ channel EAG in cervical cancer. In the future, it will be important to examine the binding of curcumin and tanshinone IIA to this channel.

## 5. Conclusions

Our findings indicate that a variety of phytochemicals, including the compounds digoxin, tanshinone IIA, DHM; and fractions of turmeric, ginger, and pomegranate inhibit the growth of cervical cells derived from precancerous lesions containing episomal or integrated HPV DNA.

Among these, curcumin and tanshinone IIA were the most active and relatively nontoxic, single agents. Tanshinone IIA and curcumin appear to target cervical cancer through multiple pathways. RT PCR analysis shows that tanshinone IIA activates the expression of p53 and suppresses HPV16 E1, E2, E4, E6 and E7 viral transcripts in W12 type 1 and 2 integrant clones. Our results indicate curcumin alters the expression of cell cycle control, apoptotic, anti-apoptotic, and immune response genes and synergizes with tanshinone IIA in HeLa cells. Further, our molecular docking results suggest that curcumin and tanshinone IIA may bind to the Na^+^/K^+^-ATPase; curcumin binds with high affinity, comparable to that of digitoxin, while tanshinone IIA shows weaker binding affinity. Insights in bioelectric signaling suggest that, by altering the activity of the Na^+^/K^+^-ATPase, compounds, such as curcumin and tanshinone IIA, either alone or in combination, could potentially modulate the development and progression of cancer.

## Figures and Tables

**Table 1:** Growth inhibitory activity of phytochemicals on cervical and colon cancer; and W12 cells.

**A** **Growth inhibitory activity of phytochemicals on cervical and colon cancer cells**				
**cells**			**HeLa**	**HT29**
**compound (µg/mL)**				
tanshinone IIA			1.33 (4.52) *	1.27 (4.32) *
carnosic acid			2.03 (6.11) *	2.13 (6.41) *
digoxin			0.12 (0.15) *	0.13 (0.17) *
**fraction (µg/mL)**				
turmeric (95% curcuminoids)			6.5	2.5
ginger			9.8	7.2
pomegranate			20	40
milk thistle			90	39
kava			45	75
boswellia			>100	45
**B** **Growth inhibitory activity of phytochemicals on W12 cells**				
**W12 clone**	**20850**	**20822**	**20862**	**20861**
**compound (µg/mL)**				
tanshinone IIA	2.3 (7.8) *	2.0 (6.8) *	3.1 (10.6)*	1.4 (4.8) *
DHM	24.7 (89.4) *	10.2 (36.9) *	12.8 (46.3) *	12.4 (44.9) *
actein	41.7 (61.6) *	55.7 (82.3) *	36.3 (53.6) * *	37.9 (56.0) *
carnosic acid	13.0 (39.1) *	10.7 (32.1) *	10.0 (30.0) *	14.1 (42.4) *
**fraction (µg/mL)**				
turmeric (95% curcuminoids)	4.7	2.8	2.0	1.6
ginger	13.7	11.6	21.6	15.5
pomegranate	25.4 **	20	28 **	10

* Concentration in µM in parentheses; ** estimates; Cells were exposed to increasing concentrations of agents for 120 h and the number of viable cells determined by the MTT assay.

**Table 2:** Cell Cycle Genes Altered by Curcumin.

Gene Identifier	Gene Name	SYMBOL	Ratio	*p*-Value
NM_005445	Structural maintenance of chromosomes 3	SMC3	−3.89	0.0136
NM_001042749	Stromal antigen 2	STAG2	−3.56	0.0463
NM_005030	Polo-like kinase 1 (Drosophila)	PLK1	2.01	0.0293
NM_002093	Glycogen synthase kinase 3 beta	GSK3B	2.09	0.0006
NM_001950	E2F transcription factor 4, p107/p130-binding	E2F4	2.37	0.0042
NM_005862	Stromal antigen 1	STAG1	2.37	0.0277
NM_003443	Zinc finger and BTB domain containing 17	ZBTB17	2.48	0.0115
NM_001254	Cell division cycle 6 homolog (S. cerevisiae)	CDC6	2.68	0.0000421
NM_004064	Cyclin-dependent kinase inhibitor 1B (p27, Kip1)	CDKN1B	2.77	0.0457
NM_001951	E2F transcription factor 5, p130-binding	E2F5	2.94	0.0374
NM_004153	Origin recognition complex, subunit 1-like (yeast)	ORC1L	3.10	0.0007
NM_005359	SMAD family member 4	SMAD4	3.49	0.0129
NM_005983	S-phase kinase-associated protein 2 (p45)	SKP2	5.03	0.0076
NM_000051	Ataxia telangiectasia mutated	ATM	15.43	0.0188
NM_016263	Fizzy/cell division cycle 20 related 1 (Drosophila)	CDC20	19.4	0.0476
NM_138292	Ataxia telangiectasia mutated	ATM	35.94	0.00000342

HeLa cells were treated with Curcumin (32 µM) and collected for RNA extraction at 8 h. Samples were prepared for gene expression analysis, as described in Section 2.7.

## Data Availability

The data presented in this study are available on request from the corresponding author, but they are not publicly available due to the requirements of ongoing research.

## Supplementary Materials

The following supporting information can be downloaded at: https://www.mdpi.com/article/10.3390/v17010055/s1, Figures S1: Structures of curcumin, tanshinone IIA and dihydromethysticin and HPLC of turmeric (95% curcuminoids); Figure S2: Growth inhibitory activity of combinations of herbal components: turmeric (95% curcuminoids) and A,B) ginger; C,D) carrageenan, on HeLa cells; Figure S3: Molecular docking of tanshinone IIA and curcumin to Na^+^K^+^-ATPase; and S2; Tables S1: Primer sequences used in RT-PCR [50,51,52,53,54]; Table S2: Effect of Tanshinone IIA on the relative level of HPV16 and p53 mRNAs in W12 cells; Table S3: Statistical analysis for Figure 4C,D; Table S4: Statistical analysis for Figure 5A (Table S2); Table S5: Statistical analysis for Figure 5C; Table S6. Technical Data Sheet for Turmeric Rhizome; Table S7. Technical Data Sheet for Kava Kava; Table S8: Technical Data Sheet for Milk Thistle Seed; Table S9: Technical Data Sheet for Boswellia Resin; Table S10: Technical Data Sheet for Blueberry; Table S11: Technical Data Sheet for Tanshinone IIA; Table S12: Technical Data Sheet for Dihydromethysticin; Table S13: Technical Data Sheet for Carnosic acid; Table S14: Technical Data Sheet for Digoxin; Table S15. Technical Data Sheet for Actein.

## Abbreviations

ANOVA: analysis of variance; CS, cardiotonic steroid; CI, combination index; cur, curcumin; DMSO, dimethylsulfoxide; EGCG, epigallocatechin gallate; EtOH, ethanol; H_2_O, water; NIGMS, National Institute of General Medical Sciences; ROS, reactive oxygen species; SE, standard error; TOL, targeted osmotic lysis; VGSC, voltage gated sodium channels; Vmem, membrane voltage potential.
