# Supplementary material for: Plant Compounds Inhibit the Growth of W12 Cervical Precancer Cells Containing Episomal or Integrant HPV DNA; Tanshinone IIA Synergizes with Curcumin in Cervical Cancer Cells
Source: Viruses. 2024 Dec 31;17(1):55. doi: 10.3390/v17010055 (PMC11768664; doi:10.3390/v17010055)
Supplement: Supplementary file 1 [file viruses-17-00055-s001.zip › Supplementary Material-1-e.pdf]

Supplementary Material

Title: Plant compounds inhibit the growth of cervical precancer cells containing episomal or integrant HPV DNA; tanshinone IIA synergizes with curcumin in cervical cancer cells

1. Combinations of turmeric (95% curcuminoids) plus ginger or carrageenan

1.1 Combination of turmeric (95% curcuminoids) plus ginger

. When increasing concentrations of both turmeric (95% curcuminoids) and ginger were combined, at a dose of ginger 0.8 μg/ml, the percent viable cells decreased from 92.64% with ginger alone to 82.29% with curcurmin 0.2 μg/ml, to 73.22% with curcumin 0.8 μg/ml, to 55.37% with curcumin 2.0 μg/ml, to 11.40% with curcumin 10 μg/ml ($p<0.01$) ((Fig. 5 A,B). Thus, curcumin enhances the growth inhibitory effect of ginger on the human cervical cancer cell line Hela.

1.2 Combination of turmeric (95% curcuminoids) plus carrageenan

When increasing concentrations of both turmeric (95% curcuminoids) and carrageenan were combined, at a dose of carrageenan 2 μM, the percent viable cells decreased from 48.5% with carrageenan alone to 37.2% with curcurmin 0.1 μg/ml, to 49.9% with curcumin 0.4 μg/ml, to 32.8% with curcumin 1.6 μg/ml, to 19.6% with curcumin 4 μg/ml ($p<0.01$) (Fig. 5 C,D) .Thus, curcumin enhances the growth inhibitory effect of carrageenan on the human cervical cancer cell line Hela.

Supplementary Material Figures

Figure S1. Structures of curcumin, tanshinoone IIA and dihydromethysticin and HPLC of turmeric (95% curcuminoids)

A. Curcumin
B. Tanshinine IIA
C. Dihydromethysticin
D. HPLC of turmeric (95% curcuminoids)

A)

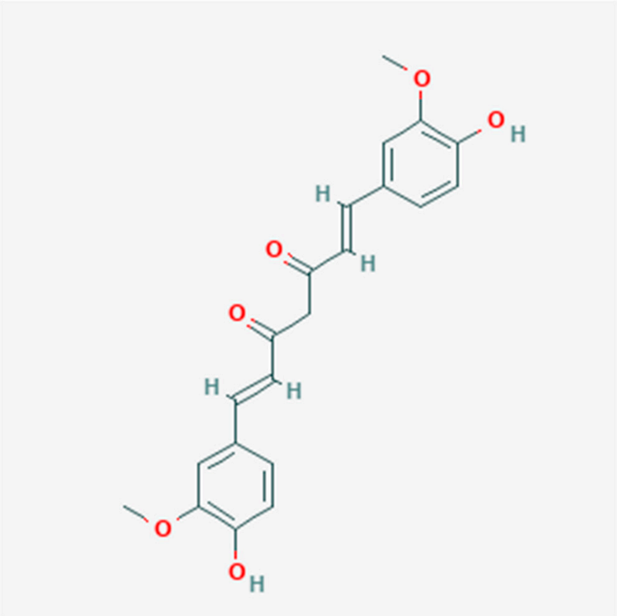

B)

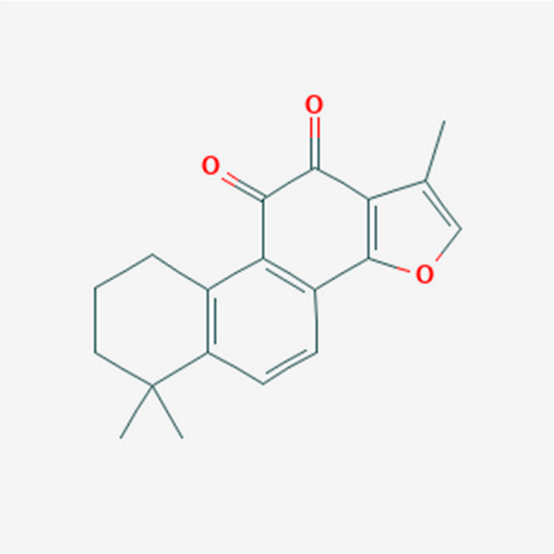

C)

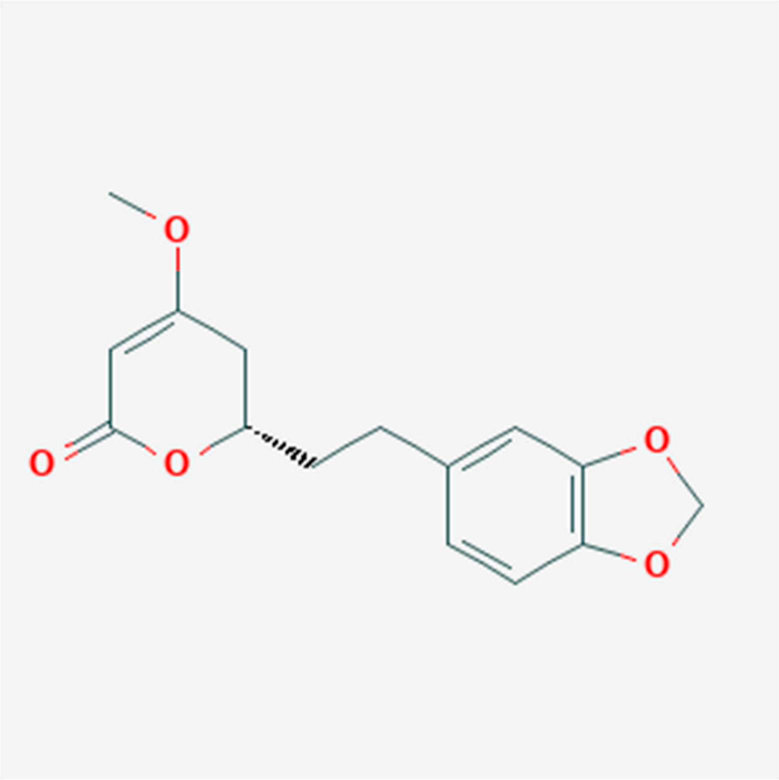

D)

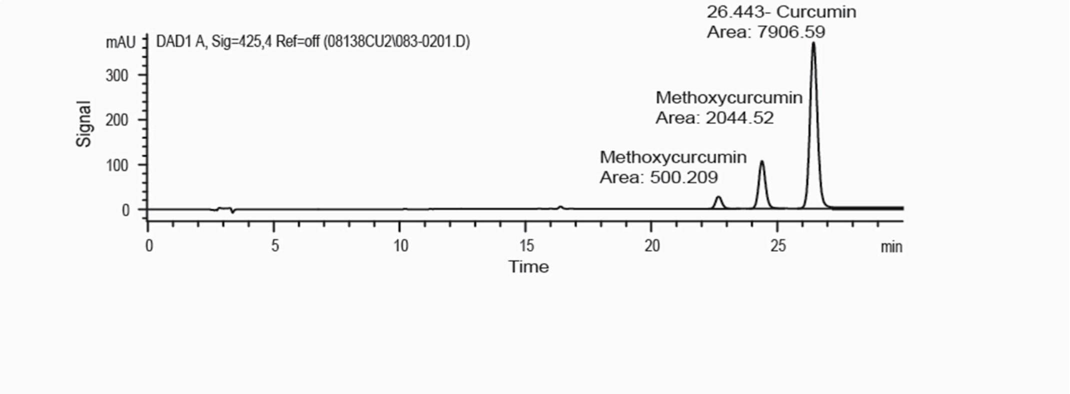
26.443- Curcumin
Area: 7906.59
mAU
DAD1 A, Sig=425,4 Ref=off (08138CU2\083-0201.D)
300
200
100
0
Signal
Methoxycurcumin
Area: 2044.52
Methoxycurcumin
Area: 500.209
0
5
10
15
20
25
min
Time

Figure S2. Growth inhibitory activity of combinations of herbal components: turmeric (95% curcuminoids) and A,B) ginger; C,D) carrageenan, on Hela cells

A,B) Combination of turmeric (95% curcuminoids) plus ginger;

A) x-axis: (95% curcuminoids);;

B) x-axis; ginger (20% total pungent compounds calculated as gingerols and shogaol)

C,D) Combination of turmeric (95% curcuminoids) plus carrageenan;

C) x-axis: (95% curcuminoids);

D) x-axis; carrageenan;

We treated HeLa cells with all combinations of 4 concentrations of each of the agents tested and a solvent control [19]. Cells were exposed to increasing concentrations of agents for 96 h and the number of viable cells determined by: A,B) EZQUANT assay; C,D) MTT assay;

LSE FIG5

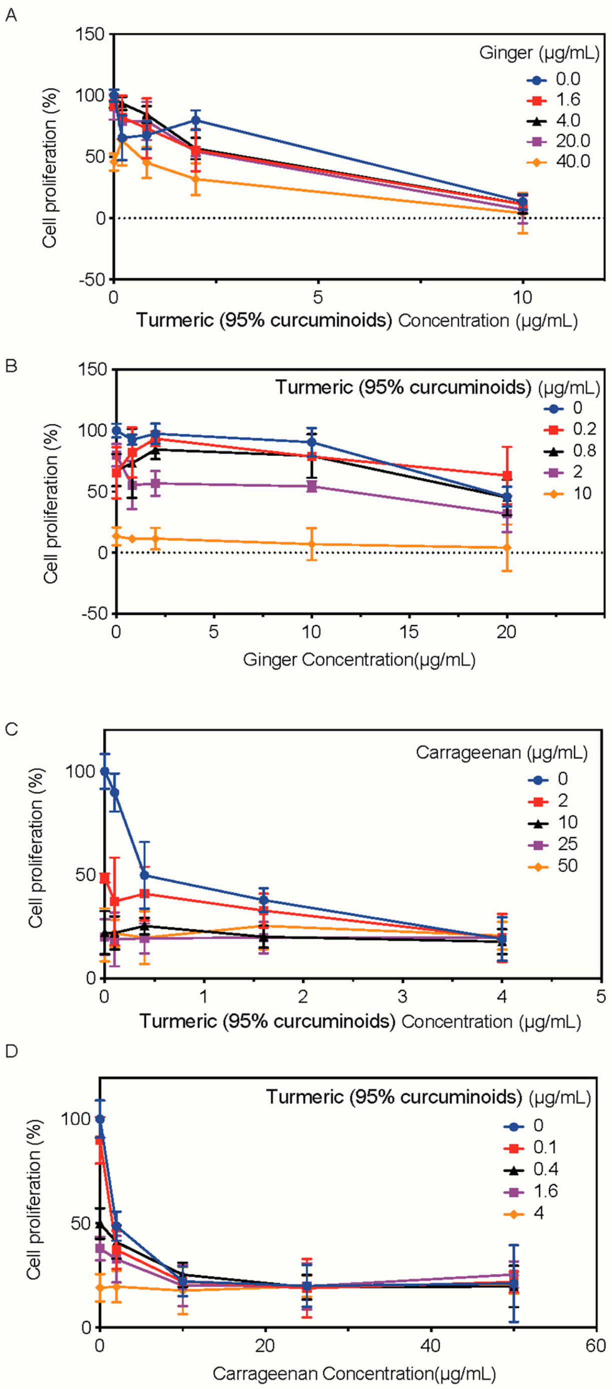

A
Cell proliferation (%)
150
100
50
0
-50
Ginger (µg/mL)
0.0
1.6
4.0
20.0
40.0
0
5
10
Turmeric (95% curcuminoids) Concentration (µg/mL)
B
Cell proliferation (%)
150
100
50
0
-50
Turmeric (95% curcuminoids) (µg/mL)
0
0.2
0.8
2
10
0
5
10
15
20
Ginger Concentration(µg/mL)
C
Cell proliferation (%)
100
50
0
Carrageenan (µg/mL)
0
2
10
25
50
0
1
2
3
4
5
Turmeric (95% curcuminoids) Concentration (µg/mL)
D
Cell proliferation (%)
100
50
0
Turmeric (95% curcuminoids) (µg/mL)
0
0.1
0.4
1.6
4
0
20
40
60
Carrageenan Concentration(µg/mL)

Figure S3. Molecular docking of tanshinone IIA and curcumin to **Na+K+ - ATPase**

A) Tanshinone IIA

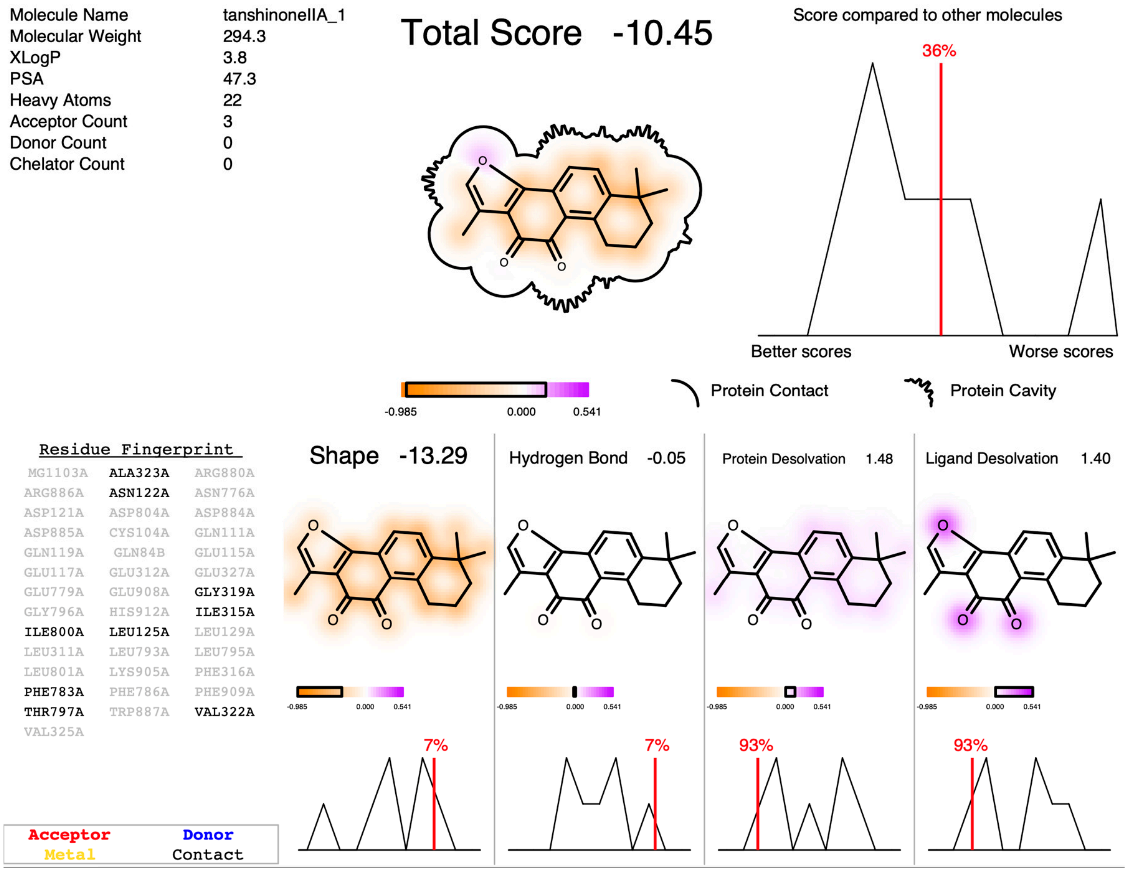

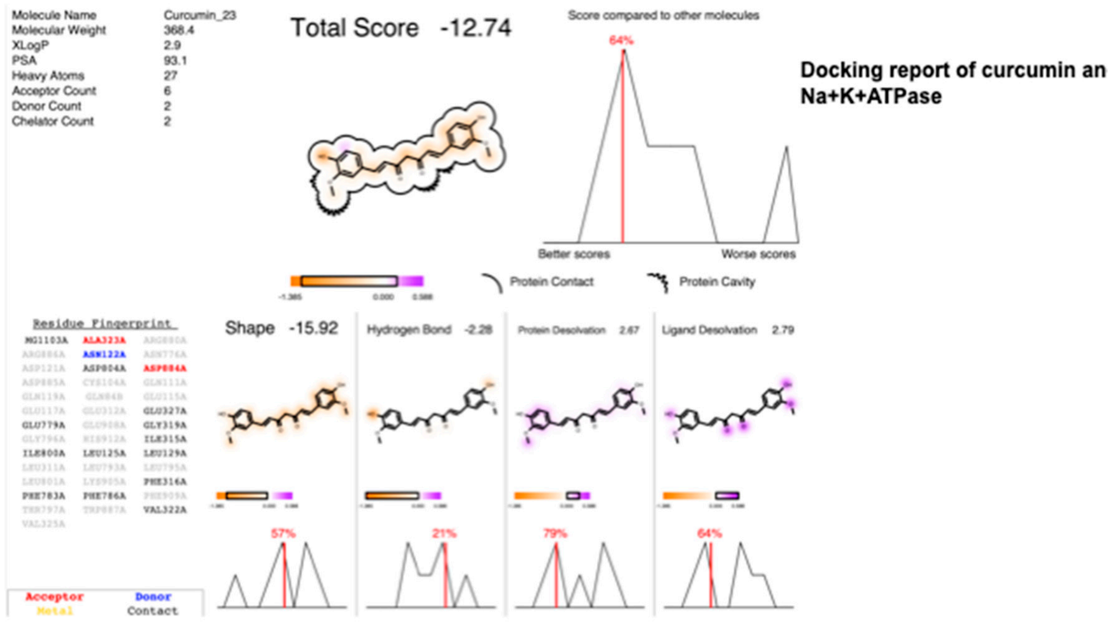

B) Curcumin

Molecule Name	Curcumin_23
Molecular Weight	368.4
XLogP	2.9
PSA	93.1
Heavy Atoms	27
Acceptor Count	6
Donor Count	2
Chelator Count	2

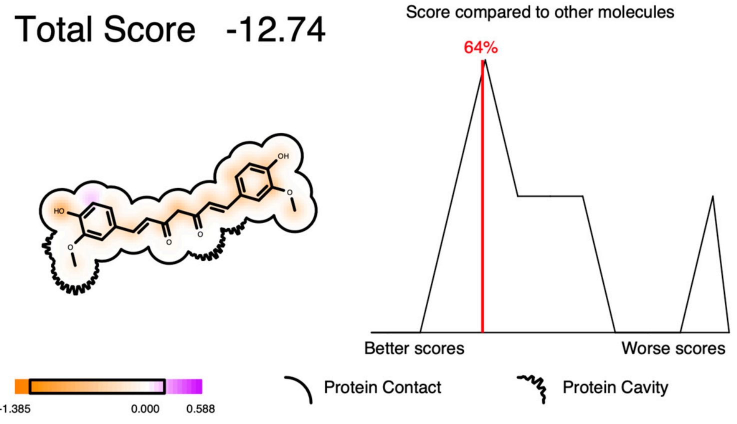

Residue Fingerprint

MG1103A	ALA323A	ARG880A
ARG886A	ASN122A	ASN776A
ASP121A	ASP804A	ASP884A
ASP885A	CYS104A	GLN111A
GLN119A	GLN84B	GLU115A
GLU117A	GLU312A	GLU327A
GLU779A	GLU908A	GLY319A
GLY796A	HIS912A	ILE315A
ILE800A	LEU125A	LEU129A
LEU311A	LEU793A	LEU795A
LEU801A	LYS905A	PHE316A
PHE783A	PHE786A	PHE909A
THR797A	TRP887A	VAL322A
VAL325A		

Acceptor	Donor
Metal	Contact

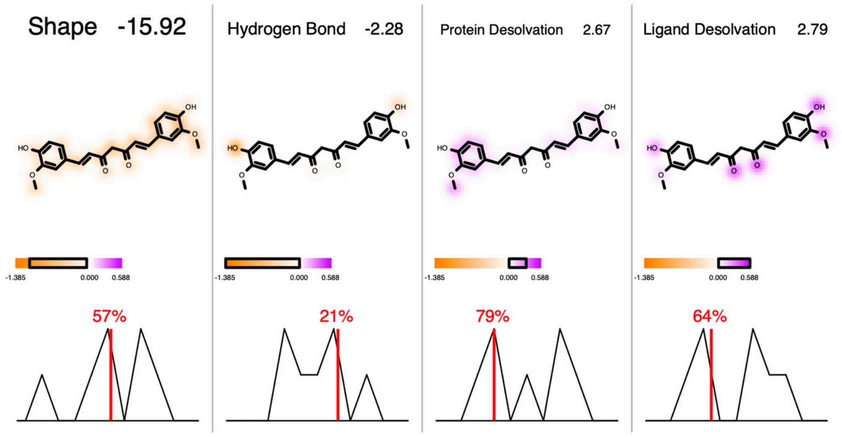

Supplementary Material Tables
Table S1. Primer sequences used in RT-PCR.

Gene name	Primer direction	Sequence	Reference
**TP53**	Forward	CACATGACGGAGGTTGTGAG	*
	Reverse	ACACGCAAATTTCCTTCCAC	
**E1**	Forward	AACGTGTTGCGATTGGTGTA	Straub et al., 2014
	Reverse	TACGCAATTTTGGAGGCTCT	
**E2**	Forward	TGGAAGTGCAGTTTGATGGA	Straub et al., 2014
	Reverse	CCGCATGAACTTCCCATACT	
**E4**	Forward	GACTATCCAGCGACCAAGATCAG	Egawa et al., 2017
	Reverse	CTGAGTCTCTGTGCAACAACTTAGTG	
**E6**	Forward	CTGCAATGTTTCAGGACCCA	[13]
	Reverse	TCATGTATAGTTGTTTGCAGCTCTGT	
**E7**	Forward	AAGTGTGACTCTACGCTTCGGTT	Kalantari et al., 2008
	Reverse	GCCCATTAACAGGTCTTCCAAA	
**P21**	Forward	TGGAGACTCTCAGGGTCGAAA	Al-Haj et al., 2012
	Reverse	AGTTGAGAATGAAGGTGGATGA	
**MDM2**	Forward	CAGGAGACGTGAAGATGCTG	IDT**
	Reverse	AGTTGAGAATGAAGGTGGATGA	
**GAPDH**	Forward	CAGCCTCAAGATCATCAGCA	*
	Reverse	GTCTTCTGGGTGGCAGTGAT	
**b-actin**	Forward	TGCCGACAGGATGCAGAAG	Ma et al., 2014
	Reverse	CTCAGGAGGAGCAATGATCTTGA	
**HPV16E5**	Forward	GTCTGTGTCTACATACACATCATTAATAC TATTG	Scott et al., 2020
	Reverse	GTAATTAAAAAGCGTGCATGTGTATGT	

*The mRNA sequences were obtained from the public GeneBank database (www.ncbi.nlm.nih.gov), and the primers were designed using Primer 3 software obtained from The Massachusetts Institute of Technology (frodo.wi.mit.edu/cgi-bin/primer3/primer3_www.cgi).
** The primer sequences were designed by Integrated DNA Technologies, Inc. (IDT).

Table S2. Effect of Tanshinone IIA on the relative level of Hpv16 and p53 mRNAs in W12 cells.

**Experiments**		
**W12 cells type**	**type 1**	**type 2**
**W12 cells clone number**	**20822**	**20862**
Relative level of mRNA Gapdh	1+/-0.14	1+/-0.13
E1	0.46+/-0.05	0.46+/-0.19
E2	0.44+/-0.02	0.54+/-0.11
E4	0.78+/-0.03	0.51+/-0.15
E6	0.84+/-0.17	0.72+/-0.09
E7	0.78+/-0.07	0.66+/-0.12
p53*	1.96+/+.04	2.53+/-0.18

The experiments were performed on different clones of W12 cells. W12 cells were treated with Tanshinone IIA at 5 uM for 24 hours; extracts were prepared and analyzed by Real-time RT-PCR, as described in Materials and Methods. Fold change indicates relative expression in Tanshinone IIA treated versus control cells.
*separate experiment

Statistical Analysis of the Figures

In the interest of space, we have included portions of the statistical analyses of some figures, as examples, and can provide complete analyses upon request.
Non-significant: yellow

Table S3. Statistical analysis for Figure 4 C,D.

Growth inhibitory activity of combinations of phytochemicals: Turmeric (95% curcuminoids) plus tanshinone IIA on HeLa cells;

	Mean1	Mean2	Mean3	Mean4	Mean5
**Tashinone IIA**	0	0.2	0.8	2	8

ANOVA based T-test									
T(Mean1-Mean2)	T(Mean1-Mean3)	T(Mean1-Mean4)	T(Mean1-Mean5)	T(Mean2-Mean3)	T(Mean2-Mean4)	T(Mean2-Mean5)	T(Mean3-Mean4)	T(Mean3-Mean5)	T(Mean4-Mean5)
9.244	17.759	17.862	17.974	8.515	8.617	8.730	0.103	0.215	0.113
11.379	18.900	18.718	19.100	7.521	7.339	7.721	0.181	0.201	0.382
13.381	21.872	21.754	22.195	8.490	8.373	8.813	0.118	0.323	0.441
11.849	20.550	21.759	20.099	8.701	9.910	8.250	1.209	0.450	1.660
1.283	6.341	6.752	6.757	5.058	5.469	5.474	0.411	0.416	0.005

P value of ANOVA based T-test (2 tails)									
**T(Mean1-Mean2)**	**T(Mean1-Mean3)**	**T(Mean1-Mean4)**	**T(Mean1-Mean5)**	**T(Mean2-Mean3)**	**T(Mean2-Mean4)**	**T(Mean2-Mean5)**	**T(Mean3-Mean4)**	**T(Mean3-Mean5)**	**T(Mean4-Mean5)**
5.11789E-14	1.38764E-28	9.76622E-29	6.65697E-29	1.24972E-12	7.96087E-13	4.85857E-13	0.918379099	0.830012622	0.910637472
5.30154E-18	3.02019E-30	5.49025E-30	1.56431E-30	9.69837E-11	2.13387E-10	4.03802E-11	0.856729397	0.841440061	0.703609347
1.46564E-21	2.78945E-34	3.95737E-34	1.07372E-34	1.39139E-12	2.32972E-12	3.37331E-13	0.906769854	0.747478511	0.660724239
7.43366E-19	1.54711E-32	3.90001E-34	6.33781E-32	5.52644E-13	2.82095E-15	3.98529E-12	0.230316815	0.653681207	0.101124138
0.203505383	1.55371E-08	2.69861E-09	2.64257E-09	2.91986E-06	5.70493E-07	5.59344E-07	0.682035832	0.678465295	0.996106365

Table S4. Statistical analysis for Figure 5A (Table S2):
RT-PCR analysis of RNA obtained after treating W12 cells with tanshinone IIA;

mRNA	p value
GAPDH	1.00
HPV-E1	1.00
HPV-E2	0.1963
HPV-E4	0.0378
HPV-E6	0.3407
HPV-E7	0.2090
p53	0.0059

Table S5. Statistical analysis for Figure 5C:

**Genes significantly altered by curcumin** (DMSO-8 vs Cur-8)	
**Pro-Apoptotic genes;**	**P value**
PRUNE2	0.0072
PPP2CA	0.0001
MAD1L1	0.0021
HMGB1	0.1154
**Anti-Apoptotic genes;**	**P value**
PEG10	0.0002
NOTCH1	0.0518
USP47	0.0066
NR4A2	0.0042

References

Al-Haj L, lackshear PJ, Khabar KSA,. Regulation of p21/CIP1/WAF-1 Mediated Cell-Cycle Arrest by RNase L and Tristetraprolin, and Involvement of AU-rich Elements. Nucleic Acids Res. 2012, 40, 7739-7752.

Einbond L.S., Shimizu M, Nuntanakorn P., Seter C., Cheng R., Jiang B. et al., 2006. Actein and a fraction of black cohosh potentiate antiproliferative effects of chemotherapy agents on human breast cancer cells. Planta Med. 72, 1200-1206.

Egawa N, Wang Q, Griffin HM, Murakami I, Jackson D, Mahmood R, Doorbar J. HPV16 and 18 genome amplification show different E4-dependence, with 16E4 enhancing E1 nuclear accumulation and replicative efficiency via its cell cycle arrest and kinase activation functions. PLoS Pathog. 2017, 13, e1006282

Kalantari M, Lee D., Calleja-Macias I.E., Lambert P.F., Bernard H.U. Effects of cellular differentiation, chromosomal integration and 5-aza-2'-deoxycytidine treatment on human papillomavirus-16 DNA methylation in cultured cell lines. Virology 2008, 374, 292-303.

Ma D, Jiang C, Hu X, Li Q, Li, T, Yang Y, Li Q. Methylation Patterns of the IFN-γ Gene in Cervical Cancer Tissues. Sci Rep. 2014; 4: 6331.

Scott MI, Woodby BI, Ulicny J, Raikhy G, Orr AW, Songock WK, Bodily JM. Human Papillomavirus 16 E5 Inhibits Interferon Signaling and Supports Episomal Viral Maintenance. J Virol 2020, 94, e01582-19.

Straub E., Dreer M., Fertey J., Iftner T., Stubenrauch F.
The viral E8^E2C repressor limits productive replication of human papillomavirus 16.
J Virol. 2014, 88, 937-947.
