# Supplementary material for: Plant Compounds Inhibit the Growth of W12 Cervical Precancer Cells Containing Episomal or Integrant HPV DNA; Tanshinone IIA Synergizes with Curcumin in Cervical Cancer Cells
Source: Viruses. 2024 Dec 31;17(1):55. doi: 10.3390/v17010055 (PMC11768664; doi:10.3390/v17010055)
Supplement: Supplementary file 1 [file viruses-17-00055-s001.zip › Supplementary Material-2-Table S6-15-e.pdf]

Table S6. Technical Data Sheet for Turmeric Rhizome

Givaudan

Technical Data Sheet

EC840501 **Turmeric rhizome PE 95% curcuminoids HPLC Granular**

Product information

Sensory profile	Typical
Color	dark yellow
Appearance	granular
Source used	Turmeric (Curcuma longa L.)
Part used	Rhizomes
Process used	Extraction, spray drying
Extraction solvent used	Ethyl acetate (100%)
Purification solvent used	Ethyl acetate
Native extract ratio (NER)	22-60/1
Equivalence statement	1 kg of product is made from an average of 41 kg of dry turmeric rhizomes.

Parameters for positive release

Analysis	Specification Limits	Method
Appearance	Conform	Visual (CQ-MO-148)
Sensory evaluation	Conform	Sensory (CQ-MO-148)
Curcumin	77.0 - 87.0 %	HPLC (LA-15-062)
Identification test	Conform	HPLC (LA-15-062)
Bulk density	0.50 - 0.90 g/ml	Graduated cylinder (CQ-MO-257)
Loss on drying	<= 8.0 %	I.R. balance (CQ-MO-018)
Particles <20 Mesh (850 micron)	>= 95.0 %	Sieve (CQ-MO-023)
Particles <100 Mesh (150 micron)	<= 20.0 %	Sieve (CQ-MO-023)
Ethyl acetate	<= 5,000 mg/kg	GC (CQ-MO-168)
Bisdemethoxycurcumin	0.7 - 3.0 %	HPLC (LA-15-062)
Demethoxy Curcumin	13.0 - 19.0 %	HPLC (LA-15-062)
Total Curcuminoids	>= 95.0 %	HPLC (LA-15-062)
Total plate count	<= 10,000 /g	ISO 4833-1: 2013
Yeasts and moulds	<= 100 /g	ISO 21527: 2008
Arsenic	<= 1.00 mg/kg	ICP (CQ-MO-247)
Cadmium	<= 1.00 mg/kg	ICP (CQ-MO-247)
Mercury	<= 0.10 mg/kg	ICP (CQ-MO-247)
Lead	<= 1.00 mg/kg	ICP (CQ-MO-247)
Heavy metal	<= 10.00 mg/kg	ICP (CQ-MO-247)-not including Chromium

Parameters not routinely tested

Analysis	Specification Limits	Method
Carbon 14	Conform	
Coliforms	<= 10 /g	ISO 4832: 2006
E. coli	Negative /g	ISO 7251: 2005
Salmonella	Negative /25g	ISO 6579-1 2017

Givaudan

Technical Data Sheet

EC840501 **Turmeric rhizome PE 95% curcuminoids HPLC Granular**

Parameters not routinely tested

Analysis	Specification Limits	Method
Bile-tolerant gram-negative bacteria	<= 100 /g	ISO 21528-2: 2017 without confirmation
Aflatoxine B1	<= 5 µg/kg	
Conjoitly aflatoxine B1,B2,G1,G2	<= 10 µg/kg	
Ochratoxin A	<= 15 µg/kg	
Pesticide Evaluation	Conform	
Benzo[a]pyrene	<= 10 µg/kg	
PAH 4	<= 50 µg/kg	

Micro Testing Principles	Low micro load, intrinsically stable. Guaranteed limits. Testing not applicable.
Heavy metals	Conform to regulatory requirements.

Storage and handling

Shelf life	728 Days
Storage conditions	Dry, preferably full, hermetically sealed
Temperature conditions	Ambient / 10-30°C (50-85°F)
Handling	Protect against light.

Miscellaneous Compliance Information

Agency(geography)	Category	Value
European Union	Contaminants Compliance	Complies with European Regulation 396/2005/EC on pesticide residue evaluation.
United States of America (USA)	Contaminants Compliance	Complies with United States Pharmacopeia (USP) General Chapter <561> on pesticide residue evaluation.
United States of America (USA)	Contaminants Compliance	Complies with United States Pharmacopeia (USP) General Chapter <467> on residual solvent evaluation.
United States of America (USA)	Regulatory Compliance Food	Complies with the US Dietary Supplement Health and Education Act of 1994.
United States of America (USA)	Regulatory Compliance Food	Complies with US Code of Federal Regulation 21 CFR 182.A (GRAS).

Givaudan

Technical Data Sheet

EC840501 **Turmeric rhizome PE 95% curcuminoids HPLC Granular**

Certifications

Certification type	Certified by/Certification N°	Facility
Certified Halal	IFANCA	South Hackensack (United States of America)
Certified Kosher Parve	KOF-K Kosher Supervision	South Hackensack (United States of America)
Certified Kosher Parve	OK Kosher Certification	Swadlincote (United Kingdom)

The above provides a summary of the certifications relevant to the product. Actual certifications may be sales order related.

Miscellaneous

Custom Tariff number 3203.00

This document is computer generated and consequently not signed.
The information contained herein is, to the best of our knowledge, true and accurate.
All information is valid until revisions are issued.
It is the customer's responsibility to ensure that the usage of the product and the levels of such usage is permitted according to the relevant laws and regulations governing the application for which the product is intended.

Givaudan Flavors Corporation 1199 Edison Drive, Cincinnati OH 45216, USA
Technical Information Services TEL: 513-948-3587 FAX:
na.document_service@givaudan.com

Issue date: 16 May 2023 - 10:57
RSBND0921623-siegelm-20230516155503

Table S7. Technical Data Sheet for Kava Kava

Givaudan

Technical Data Sheet

EC841560 | **KAVA KAVA ROOT PE 30% KAVA LACTONES HPLC**

01580

Product information

Sensory profile	Typical
Color	light yellow to tan
Appearance	powder
Source used	Kava kava (Piper methysticum G.Forst.)
Part used	Roots
Process used	Extraction, spray drying
Extraction solvent used	Ethanol (95%) / Water (5%)
Native extract ratio (NER)	5-8/1
Equivalence statement	1 kg of product is made from an average of 3.0 kg of dry kava kava roots.

Parameters for positive release

Analysis	Specification Limits	Method
Appearance	Conform	Visual (CQ-MO-148)
Sensory evaluation	Conform	Sensory (CQ-MO-148)
Identification test	Conform	HPLC (CQ-MO-027)
Bulk density	>= 0.30 g/ml	Graduated cylinder (CQ-MO-257)
Loss on drying	<= 8.0 %	I.R. balance (CQ-MO-018)
Particles <40 Mesh (400 micron)	>= 95.0 %	Sieve (CQ-MO-023)
Kavalactones	>= 30.00 %	HPLC (CQ-MO-027)
Total plate count	<= 10,000 /g	ISO 4833-1: 2013
Yeasts and moulds	<= 100 /g	ISO 21527: 2008
Ethanol	<= 5,000.0 mg/kg	GC (CQ-MO-168)
Arsenic	<= 2.00 mg/kg	ICP (CQ-MO-247)
Cadmium	<= 0.82 mg/kg	ICP (CQ-MO-247)
Mercury	<= 0.10 mg/kg	ICP (CQ-MO-247)
Lead	<= 1.00 mg/kg	ICP (CQ-MO-247)
Heavy metal	<= 10.00 mg/kg	ICP (CQ-MO-247)-not including Chromium

Parameters not routinely tested

Analysis	Specification Limits	Method
Enterobacteriaceae	<= 1,000 /g	ISO 21528-2: 2017
Coliforms	<= 10 /g	ISO 4832: 2006
E. coli	Negative /10g	ISO 7251: 2005
Salmonella	Negative /25g	ISO 6579-1 2017
Bile-tolerant gram-negative bacteria	<= 100 /g	ISO 21528-2: 2017 without confirmation
Pesticide Evaluation	Conform	
Benzo[a]pyrene	<= 10 µg/kg	
PAH 4	<= 50 µg/kg	

Givaudan

Technical Data Sheet

EC841560 **KAVA KAVA ROOT PE 30% KAVA LACTONES HPLC**

01580

Parameters not routinely tested

Analysis	Specification Limits	Method
Micro Testing Principles	Limits guaranteed, selected microbiological parameters are monitored.	
Heavy metals	Conform to regulatory requirements.	

Storage and handling

Shelf life	728 Days
Storage conditions	Dry, preferably full, hermetically sealed
Temperature conditions	Ambient / 10-30°C (50-85°F)
Handling	Protect against light.

Miscellaneous Compliance Information

Agency(geography)	Category	Value
United States of America (USA)	Contaminants Compliance	Complies with United States Pharmacopeia (USP) General Chapter <561> on pesticide residue evaluation.
United States of America (USA)	Regulatory Compliance Food	Complies with the US Dietary Supplement Health and Education Act of 1994.

Certifications

Certification type	Certified by/Certification N°	Facility
Certified Kosher Parve	KOF-K Kosher Supervision	South Hackensack (United States of America)

The above provides a summary of the certifications relevant to the product. Actual certifications may be sales order related.

Reference Application(s) and Dosage	(in %)
Water	0.25

This document is computer generated and consequently not signed.
The information contained herein is, to the best of our knowledge, true and accurate.
All information is valid until revisions are issued.
It is the customer's responsibility to ensure that the usage of the product and the levels of such usage is permitted according to the relevant laws and regulations governing the application for which the product is intended.

Givaudan Flavors Corporation 1199 Edison Drive, Cincinnati OH 45216, USA
Technical Information Services TEL: 513-948-3587 FAX:
na.document_service@givaudan.com

Table S8. Technical Data Sheet for Milk Thistle Seed

Givaudan

Technical Data Sheet

EC840533 **MILK THISTLE SEED PE 80% SILYMARIN UV**

34180

Product information

Sensory profile	Typical
Color	beige to Brownish yellow
Appearance	powder
Source used	Milk thistle (Silybum marianum (L.) Gaertn.)
Part used	Seeds
Process used	Extraction
Extraction solvent used	Acetone (80-100%) / Water (0-20%)
Native extract ratio (NER)	24-26/1
Equivalence statement	1 kg of product is made from an average of 25.0 kg of dry milk thistle seeds.

Parameters for positive release

Analysis	Specification Limits	Method
Appearance	Conform	Visual (CQ-MO-148)
Sensory evaluation	Conform	Sensory (CQ-MO-148)
Identification test	Conform	TLC (QC-SOP-018)
Bulk density	0.30 - 0.60 g/ml	Graduated cylinder (CQ-MO-257)
Loss on drying	<= 6.0 %	I.R. balance (CQ-MO-018)
Particles <80 Mesh (175 micron)	>= 100 %	Sieve (CQ-MO-023)
Hexane	<= 290 mg/kg	GC (CQ-MO-168)
Silymarin	>= 80.0 %	Spectrophotometer (CQ-MO-084)
Silybin and derivatives	>= 30.0 %	HPLC (CQ-MO-162)
Total plate count	<= 10,000 /g	ISO 4833-1: 2013
Yeasts and moulds	<= 100 /g	ISO 21527: 2008
Acetone	<= 0.5 mg/kg	GC (CQ-MO-168)
Arsenic	<= 1.00 mg/kg	ICP (CQ-MO-247)
Cadmium	<= 0.50 mg/kg	ICP (CQ-MO-247)
Mercury	<= 0.10 mg/kg	ICP (CQ-MO-247)
Lead	<= 0.50 mg/kg	ICP (CQ-MO-247)
Heavy metal	<= 10.00 mg/kg	ICP (CQ-MO-247)-not including Chromium

Parameters not routinely tested

Analysis	Specification Limits	Method
Coliforms	<= 3 /g	ISO 4831: 2006 MPN
E. coli	Negative /10g	ISO 7251: 2005
Salmonella	Negative /25g	ISO 6579-1 2017
Enterobacteriaceae	Negative /g	ISO 21528-1: 2017
Staphylococcus aureus	Negative /10g	ISO 6888-3: 2003-2004
Bile-tolerant gram-negative bacteria	<= 100 /g	ISO 21528-2: 2017 without confirmation
Aflatoxine B1	<= 5 µg/kg	

Table S8. Technical Data Sheet for Milk Thistle Seed (continued)

Givaudan

Technical Data Sheet

EC840533 **MILK THISTLE SEED PE 80% SILYMARIN UV**

34180

Parameters not routinely tested

Analysis	Specification Limits	Method
Conjoitly aflatoxine B1,B2,G1,G2	<= 10 µg/kg	
Ochratoxin A	<= 7 µg/kg	
Pesticide Evaluation	Conform	
Benzo[a]pyrene	<= 10 µg/kg	
PAH 4	<= 50 µg/kg	

Micro Testing Principles	Limits guaranteed, selected microbiological parameters are monitored.
Heavy metals	Conform to regulatory requirements.

Storage and handling

Shelf life	728 Days
Storage conditions	Dry, preferably full, hermetically sealed
Temperature conditions	Ambient / 10-30°C (50-85°F)
Handling	Protect against light.

Miscellaneous Compliance Information

Agency(geography)	Category	Value
European Union	Contaminants Compliance	Complies with European Regulation 396/2005/EC on pesticide residue evaluation.
United States of America (USA)	Contaminants Compliance	Complies with United States Pharmacopeia (USP) General Chapter <561> on pesticide residue evaluation.
United States of America (USA)	Contaminants Compliance	Complies with United States Pharmacopeia (USP) General Chapter <467> on residual solvent evaluation.
United States of America (USA)	Regulatory Compliance Food	Complies with the US Dietary Supplement Health and Education Act of 1994.

Certifications

Certification type	Certified by/Certification N°	Facility
Certified Halal	IFANCA	South Hackensack (United States of America)
Certified Kosher Parve	KOF-K Kosher Supervision	South Hackensack (United States of America)

The above provides a summary of the certifications relevant to the product. Actual certifications may be sales order related.

Table S8. Technical Data Sheet for Milk Thistle Seed (continued)

Miscellaneous

Custom Tariff number	2932.99

This document is computer generated and consequently not signed.
The information contained herein is, to the best of our knowledge, true and accurate.
All information is valid until revisions are issued.
It is the customer's responsibility to ensure that the usage of the product and the levels of such usage is permitted according to the relevant laws and regulations governing the application for which the product is intended.

Table S9. Technical Data Sheet for Boswellia Resin

Givaudan

Technical Data Sheet

EC841136 | **BOSWELLIA RESIN PE 65% BOSWELLIC ACIDS BY TITRATION**

Product information

Sensory profile	Typical
Color	beige to light brown
Appearance	powder
Source used	Boswellia (Boswellia serrata Triana & Planch.)
Part used	Resins
Process used	Extraction, spray drying
Extraction solvent used	Methanol (80-100%) / Water (0-20%)
Native extract ratio (NER)	5-10/1
Equivalence statement	1 kg of product is made from an average of 7.5 kg of dry boswellia resins.

Parameters for positive release

Analysis	Specification Limits	Method
Appearance	Conform	Visual (CQ-MO-148)
Sensory evaluation	Conform	Sensory (CQ-MO-148)
Identification test	Conform	HPTLC (QC-SOP-032)
Bulk density	>= 0.09 g/ml	Graduated cylinder (CQ-MO-257)
Loss on drying	<= 8 %	I.R. balance (CQ-MO-018)
Particles <80 Mesh (175 micron)	>= 95 %	Sieve (CQ-MO-023)
Tapped Density	>= 0.28 g/ml	Tapped Density Tester (CQ-MO-257)
Total boswellic acids	>= 65.0 %	Titration (CQ-MO-340)
Total plate count	<= 10,000 /g	ISO 4833-1: 2013
Yeasts and moulds	<= 100 /g	ISO 21527: 2008
Methanol	<= 50 mg/kg	GC (CQ-MO-168)
Arsenic	<= 1.00 mg/kg	ICP (CQ-MO-247)
Cadmium	<= 1.00 mg/kg	ICP (CQ-MO-247)
Mercury	<= 0.10 mg/kg	ICP (CQ-MO-247)
Lead	<= 0.50 mg/kg	ICP (CQ-MO-247)
Heavy metal	<= 10.00 mg/kg	ICP (CQ-MO-247)

Parameters not routinely tested

Analysis	Specification Limits	Method
Coliforms	<= 10 /g	ISO 4832: 2006
E. coli	Negative /g	ISO 7251: 2005
Salmonella	Negative /25g	ISO 6579-1 2017
Bile-tolerant gram-negative bacteria	<= 100 /g	ISO 21528-2: 2017 without confirmation
Pesticide Evaluation	Conform	
Benzo[a]pyrene	<= 10 µg/kg	
PAH 4	<= 50 µg/kg	

Table S9. Technical Data Sheet for Boswellia Resin (continued)

Givaudan

Technical Data Sheet

EC841136 **BOSWELLIA RESIN PE 65% BOSWELLIC ACIDS BY TITRATION**

Parameters not routinely tested

Analysis	Specification Limits	Method
Heavy metals	Conform to regulatory requirements.	

Storage and handling

Shelf life	728 Days
Storage conditions	Dry, preferably full, hermetically sealed
Temperature conditions	Ambient / 10-30°C (50-85°F)
Handling	Protect against light.

Miscellaneous Compliance Information

Agency(geography)	Category	Value
United States of America (USA)	Contaminants Compliance	Complies with United States Pharmacopeia (USP) General Chapter <561> on pesticide residue evaluation.
United States of America (USA)	Regulatory Compliance Food	Complies with the US Dietary Supplement Health and Education Act of 1994.

Certifications

Certification type	Certified by/Certification N°	Facility
Certified Halal	IFANCA	South Hackensack (United States of America)
Certified Kosher Parve	KOF-K Kosher Supervision	South Hackensack (United States of America)
Certified Kosher Parve	OK Kosher Certification	Swadlincote (United Kingdom)

The above provides a summary of the certifications relevant to the product. Actual certifications may be sales order related.

Miscellaneous

Custom Tariff number	2106.90

This document is computer generated and consequently not signed.
The information contained herein is, to the best of our knowledge, true and accurate.
All information is valid until revisions are issued.
It is the customer's responsibility to ensure that the usage of the product and the levels of such usage is permitted according to the relevant laws and regulations governing the application for which the product is intended.

Givaudan Flavors Corporation 1199 Edison Drive, Cincinnati OH 45216, USA
Technical Information Services TEL: 513-948-3587 FAX:
na.document_service@givaudan.com

Issue date: 16 May 2023 - 11:02
RSBND0921625-siegelm-20230516155635

Table S10. Technical Data Sheet for Blueberry

NATUREX Product Specification

BLUEBERRY 150-Z_102267 EUS **Ref : NN102267**

Description :

Blueberry 150-Z_102267 is a powder which has a blueberry flavor with a note of jam and a slight astringent and acidulous taste. It is made with blueberry juice and produced by spray drying.

Botanical name : *Vaccinium angustifolium L., Vaccinium myrtillus L.*

1kg product contains approx. 4.5 kg juice at 11°Brix.

Composition :

Blueberry juice, Maltodextrin (from maize)

Regulation status :

The raw materials used to manufacture the product are compliant with the applicable provisions set by Commission Regulation (EC) N° 1881/2006 of 19 December 2006 setting maximum levels for certain contaminants in foodstuffs. The raw materials used to manufacture the product are compliant with the applicable provisions set by the US Code of Federal regulation 40 CFR Part 180 setting tolerances and exemptions for pesticide chemical residues in food.

Specifications :

Sensory quality :

Aspect :	Spray dried powder
Color :	Violet
Flavor :	Blueberry

Analytical quality :

Particle size :	> 95 % through 0.5 mm*
Water content :	< 5.0 %
Titratable acidity (citric acid monohydrate) :	3.4 - 7.0 %
Pesticide residues :	Compliant to 40CFR180*
Pesticide residues :	Compliant to EU Regulation 396/2005*

Microbiological quality :

Total plate count :	< 5,000 cfu/g
Mould count :	< 100 cfu/g
Yeast count :	< 100 cfu/g
Enterobacteriaceae :	Not Detectable in 0.1 g
Salmonella :	Not Detectable in 50 g

**Control Plan, Analysis performed once a year.*

Naturex AG
Industriestrasse 8, CH-9220 Bischofszell – Switzerland
Phone: +41 71 424 73 00 – Fax: +41 71 424 73 90 - E-mail: group.naturex_ch@givaudan.com – Website: www.naturex.com

NATUREX

Product Specification

BLUEBERRY 150-Z_102267 EUS **Ref : NN102267**

Packaging :

Cardboard box with polyethylene bags : 20 kg net

Recommended storage conditions :

in a cool room, sheltered from light, moisture and oxygen (4 - 18°C, 60 % RH).

Best before :

36 months under the previously mentioned conditions and in its original packaging.

This specification sheet cancels and replaces all previous publications : ***August 12, 2021***

Naturex AG
Industriestrasse 8, CH-9220 Bischofszell – Switzerland
Phone: +41 71 424 73 00 – Fax: +41 71 424 73 90 - E-mail: group.naturex_ch@givaudan.com – Website: www.naturex.com

Table S11. Technical Data Sheet for Tanshinone IIA

3050 Spruce Street, Saint Louis, MO 63103, USA
Website: www.sigmaaldrich.com
Email USA: techserv@sial.com
Outside USA: eurtechserv@sial.com

Product Specification

Product Name:
Tanshinone IIA – ≥97% (HPLC)

Product Number: **T4952**
CAS Number: 568-72-9

Formula: C19H18O3
Formula Weight: 294.34 g/mol
Storage Temperature: 2 - 8 ℃

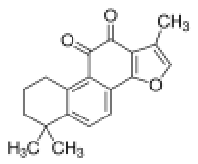

TEST	Specification
Appearance (Color)	Orange to Dark Orange to Dark Red
Appearance (Form)	Powder
IR Spectrum	Conforms to Structure
Solubility (Color)	Red to Red-Orange
Solubility (Turbidity)	Clear
5 mg/mL, MeOH	
Purity (HPLC)	≥ 97 %
(approx 98%)	
Melting Range	Conforms
209-210 Deg C	

Specification: PRD.0.ZQ5.10000103680

Sigma-Aldrich warrants, that at the time of the quality release or subsequent retest date this product conformed to the information contained in this publication. The current Specification sheet may be available at Sigma-Aldrich.com. For further inquiries, please contact Technical Service. Purchaser must determine the suitability of the product for its particular use. See reverse side of invoice or packing slip for additional terms and conditions of sale.

Table S12. Technical Data Sheet for Dihydromethysticin

Certificate of Analysis

For Laboratory Use Only

**PRODUCT NAME**	Dihydromethysticin
**PART NUMBER**	00004482
**STANDARD TYPE**	Primary (P)
**LOT NUMBER**	00004482-242
**REPORT NUMBER**	CDXA-RSS-9919-00
**SAMPLE NUMBER**	CDXP-22-00796
**DATE OF SAMPLE**	08/30/2022
**DATE OF REPORT**	09/22/2022

Structure

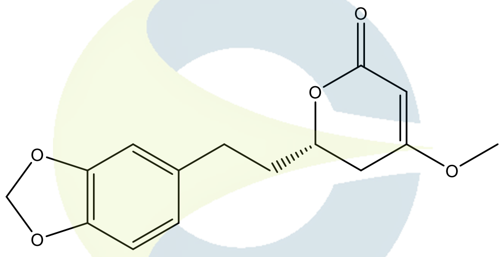

**CHEMICAL NAMES**	(6S)-6-(2-(1,3-benzodioxol-5-yl)ethyl)-5,6-dihydro-4-methoxy-2H-Pyran-2-one; 7,8-Dihydromethysticin; (S)-(+)-Dihydromethysticin
**CHEMICAL FORMULA**	$C_{15}H_{16}O_5$
**MOLECULAR WEIGHT (MW)**	276.29
**CHEMICAL FAMILY**	Kavalactones
**CAS NUMBER**	[19902-91-1]
**EC#(EINECS)**	N/A
**RTECS**	UQ0516000

ANALYTICAL RESULTS

TEST	METHOD	SPECIFICATION	RESULT
Adjusted Purity	NA	≥ 85%	99.1%
HPLC	0.700.10.2.METH22	≥ 90%	99.1%
NMR	0.700.12.4	Conforms to structure	Conforms to structure
Mass Spectrum	0.700.12.27	Conforms	Conforms
Residual Solvent*	USP <467>	NA	ND
Water	0.700.12.37	NA	ND
Appearance	NA	NA	White powder

*Testing performed at an ISO 17025 accredited subcontracted laboratory.

ADJUSTED PURITY: 99.1% IS BASED ON (100 - 0.0 SOLVENTS - 0.0 WATER) X 99.1% HPLC

STORAGE CONDITIONS

**STORAGE**	-20 °C in a dry place.
**EXPIRATION DATE**	08/2027 under the above conditions.

This document is the property of ChromaDex, Inc. and/or its relevant affiliates and contains confidential and proprietary material for the sole use of the intended recipient(s). Any review, use, distribution or disclosure by or to others is strictly prohibited.

10900 Wilshire Blvd., Suite 600, Los Angeles, CA 90024 | T: +1 949-419-0288 | F: +1 949-419-0294 | www.chromadex.com
Laboratory address: 1751 S. Fordham St., Suite 350, Longmont, CO 80503

Table S13. Technical Data Sheet for Carnosic acid

SIGMA-ALDRICH®

sigma-aldrich.com

3050 Spruce Street, Saint Louis, MO 63103, USA
Website: www.sigmaaldrich.com
Email USA: techserv@sial.com
Outside USA: eurtechserv@sial.com

Product Specification

Product Name:
Carnosic acid from Rosmarinus officinalis – ≥91%, powder

**Product Number:**	**C0609**
CAS Number:	3650-09-7
MDL:	MFCD02259459
Formula:	C20H28O4
Formula Weight:	332.43 g/mol
Storage Temperature:	-20 ℃

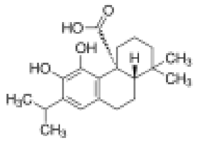

**TEST**	**Specification**
Appearance (Color)	Faint Yellow to Dark Yellow
Appearance (Form)	Powder
Proton NMR spectrum	Conforms to Structure
EmM in MeOH	1.50 - 2.00
Wavelength Lambda max	284 - 286 nm
Purity (HPLC)	≥ 91 %

Specification: PRD.1.ZQ5.10000045550

Sigma-Aldrich warrants, that at the time of the quality release or subsequent retest date this product conformed to the information contained in this publication. The current Specification sheet may be available at Sigma-Aldrich.com. For further inquiries, please contact Technical Service. Purchaser must determine the suitability of the product for its particular use. See reverse side of invoice or packing slip for additional terms and conditions of sale.

Table S14. Technical Data Sheet for Digoxin

Specification Sheet

**Product Name**	Digoxin analytical standard
**Product Number**	D6003
**Product Brand**	SIAL
**CAS Number**	20830-75-5
**Molecular Weight**	780.94

TEST	SPECIFICATION
APPEARANCE (COLOR)	White to Off White
APPEARANCE (FORM)	Powder
PURITY (HPLC AREA%)	2: 95.0%
SOLUBILITY (COLOR)	Colorless
SOLUBILITY (TURBIDITY)	Clear
SOLUBILITY (METHOD) INFRARED	0.5 % in methanol:methylene chloride 1:1 (v/v)
SPECTRUM RECOMMENDED	CONFORMS TO STRUCTURE
RETEST PERIOD	48MONTHS

Table S15. Technical Data Sheet for Actein

Planta Analytica

ABSTRACT

Home / Uncategorized / Actein

ACTEIN

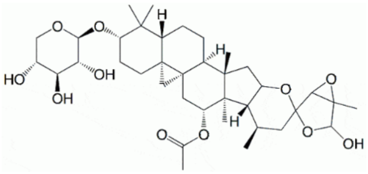

Actein (CAS 18642-44-9) and 27-Deoxyactein are the major triterpene glycosides isolated from the roots and rhizomes of black cohosh. Black cohosh (*Cimicifuga racemosa*) is a major product in chinses traditional medicine. Many of these compounds are physiologically active and possess a broad range of medico-biological action. *C. racemosa* has been traditionally exploited as an alternative therapy for the improvement of various disorders, including breast cancer and osteoporosis. Recent research indicates these triterpene glycosides may be an effective treatment for postmenopausal syndrome. Preclinical and clinical data have supported the CNS activity associated with the alleviation of post-menopausal symptoms. 27-Deoxyactein and actein have also shown efficacy in treatment of osteoporosis.

Category: Uncategorized

COMPOUND DETAILS

CAS	*18642-44-9*
Molecular Weight	*676.85*
Chemical Formula	*C37H56O11*
IUPAC	*[(1S,1'R,2S,3'R,4R,4'R,5R,5'R,6'R,10'S,12'S,13'S,16'R,18'S,21'R)-2-hydroxy-1,4',6',12',17',17'-hexamethyl-18'-[(2S,3R,4S,5R)-3,4,5-trihydroxyoxan-2-yl]oxyspiro[3,6-dioxabicyclo[3.1.0]hexane-4,8'-9-oxahexacyclo[11.9.0.01,21.04,12.05,10.016,21]docosane]-3'-yl] acetate*
Synonyms	*Shengmating*
SMILES	*CC1CC2(C3C(O3)(C(O2)O)C)OC4C1C5(C(CC67CC68CCC(C(C8CCC7C5(C4)C)(C)C)OC9C(C(C(CO9)O)O)O)OC(=O)C)C*
Purity	*98%+*
Storage Temperature	*Below -18 degrees C*
Storage Conditions	*Dry, freezer*
